# Chemical Constituents of the Leaves of *Campanula takesimana* (Korean Bellflower) and Their Inhibitory Effects on LPS-induced PGE_2_ Production

**DOI:** 10.3390/plants9091232

**Published:** 2020-09-18

**Authors:** Yutong Qi, Se-In Choi, So-Ri Son, Hee-Soo Han, Hye Shin Ahn, Yu-Kyong Shin, Sun Hee Lee, Kyung-Tae Lee, Hak Cheol Kwon, Dae Sik Jang

**Affiliations:** 1 Department of Life and Nanopharmaceutical Sciences, Graduate School Kyung Hee University, Seoul 02447, Korea; qiyutong9675@gmail.com (Y.Q.); sae_in@daum.net (S.-I.C.); allosori@khu.ac.kr (S.-R.S.); heesu3620@daum.net (H.-S.H.); ktlee@khu.ac.kr (K.-T.L.); 2 Department of New Material Development, COSMAX BIO, Seongnam 13486, Korea; hsahn@cosmax.com (H.S.A.); ykshin@cosmax.com (Y.-K.S.); bt-shlee@cosmax.com (S.H.L.); 3 Natural Product Informatics Research Center, Korea Institute of Science and Technology (KIST) Gangneung Institute, Gangneung 25451, Korea; hkwon@kist.re.kr

**Keywords:** *Campanula takesimana*, Campanulaceae, campanulalignans, phenolic compounds, PGE_2_ production

## Abstract

*Campanula takesimana* Nakai (Campanulaceae; Korean bellflower) is one of the endemic herbs of Korea. The plant has been used as traditional medicines for treating asthma, tonsillitis, and sore throat in Korea. A hot water extract of the leaves of *C. takesimana* exhibited a significant inhibitory effect on lipopolysaccharide (LPS)-stimulated prostaglandin E_2_ (PGE_2_) production. Repetitive chromatographic separation of the hot water extract led to the isolation of three new neolignan glucosides, campanulalignans A–C (**1**–**3**), with 15 known compounds (**4**–**18**). The structures of new compounds **1**–**3** were elucidated by analyzing nuclear magnetic resonance (NMR) spectroscopic data, along with high resolution quadrupole time of flight mass (HR-Q-TOF-MS) spectrometric data. Among the isolates, simplidin (**7**), 5-hydroxyconiferaldehyde (**11**), icariside F_2_ (**12**), benzyl-*α*-l-arabinopyranosyl-(1″→6′)-*β*-d-glucopyranoside (**13**), and kaempferol 3-*O*-*β*-d-apiosyl (1→2)-*β*-d-glucopyranoside (**15**) were isolated from the Campanulaceae family for the first time. The isolates (**1**, **2**, and **4**–**18**) were assessed for their anti-inflammatory effects on LPS-stimulated PGE_2_ production on RAW 264.7 cells. 7*R*,8*S*-Dihydrodehydrodiconiferyl alcohol (**5**), 3′,4-*O*-dimethylcedrusin 9-*O*-*β*-glucopyranoside (**6**), pinoresinol di-*O*-*β*-d-glucoside (**8**), ferulic acid (**10**), 5-hydroxyconiferaldehyde (**11**), and quercetin (**18**) showed significant inhibitory effects on LPS-stimulated PGE_2_ production.

## 1. Introduction

*Campanula takesimana* Nakai (Campanulaceae), known as Korean bellflower, is a native herb of Korea, growing on Ulleng island. The leaves and stems of *C. takesimana* are shiny, and the flowers have spots on a pale purple background. It is clearly distinguished from *C. punctata*, commonly distributed in Korea, China, and Japan, by its smaller overall size and lighter purple color of flower [1]. Because it has long flowering period and shape of flower, this plant has been commonly cultivated for a decorative plant. Besides the value of horticulture, roots of *C. takesimana* have been used for herbal remedies for asthma, tonsillitis, and sore throat in traditional Korean medicine [2].

It was reported that the *n*-butanol and ethyl acetate fractions of *C. takesimana* exhibit free radical-scavenging, tyrosinase inhibition, and superoxide dismutase (SOD)-like activities [2]. Although extensive research has been done on the pharmacological activities and/or phytochemical constituents of the plants belonging to family Campanulaceae, yet only few studies have been conducted on the genus *Campanula*, despite their frequent traditional use [3]. Moreover, chemical constituents of *C. takesimana* have never been reported from previous studies.

As part of our continuing project to search for bioactive compounds with anti-inflammatory activities and identify chemical constituents of higher plants, we observed that a hot water extract from the leaves of *C. takesimana* showed a significant inhibitory effect on lipopolysaccharide (LPS)-stimulated prostaglandin E_2_ (PGE_2_) production in RAW 264.7 macrophages. LPS is one of the most potent activators of macrophages, and LPS-stimulated macrophages and monocytes are known to produce inflammatory mediators. In this regard, inflammatory mediators, such as PGE_2_, nitric oxide (NO), and numerous pro-inflammatory cytokines released by the activated macrophages are important targets for the treatment of inflammation [4]. Thus, compounds that can regulate the production of pro-inflammatory mediators like PGE_2_ may be promising therapeutic agents for the treatment of various inflammatory diseases.

In the present research, we concentrated on isolating secondary metabolites from the hot water extract and identifying the active constituents. Three new neolignan glucosides (**1**–**3**) and 15 known compounds (**4**–**18**) were identified by repetitive chromatographic separation of the hot water extract. The structure of the new compounds (**1**–**3**) was elucidated by analysis of one-dimensional (1D) and two-dimensional (2D) nuclear magnetic resonance (NMR) spectroscopic and high resolution quadrupole time of flight mass (HR-Q-TOF-MS) spectrometric data. The structures of the known compounds were identified in comparison with previously published values. Thereafter, the isolates were assessed for their inhibitory effects on the production of the pro-inflammatory mediator, PGE_2_, in LPS-stimulated RAW264.7 macrophages. We describe in this paper the isolation of the secondary metabolites from the leaves of *P. japonicus*, structure elucidation of the three new neolignan glucosides (**1**–**3**), and inhibitory effects of the isolates on PGE_2_ production.

## 2. Results

### 2.1. Structure Elucidation of New Compounds 1–3

**Figure 1:**
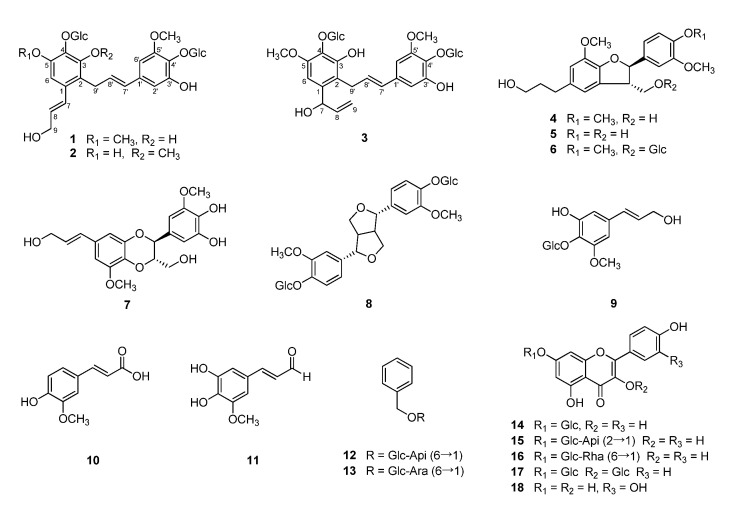
Structures of compounds **1**–**18** isolated from *Campanula takesimana*.

By repetitive chromatographic separation of the hot water extract, three new neolignan glucosides (**1**–**3**) were isolated, along with 15 known compounds, including five lignans (**4**–**8**), five phenolic compounds (**9**–**13**), and five flavonoids (**14**–**18**), in the present research (Figure 1).

Compound **1** was isolated as a pale yellow powder. Based on its molecular ion peak [M−H]^−^ at *m*/*z* 697.2360 (calcd for C_32_H_41_O_17_, 697.2344) in negative mode of HR-Q-TOF-MS (Figure S1), the molecular formula of **1** was established as C_32_H_42_O_17_. The infrared absorption (IR) spectrum displayed absorption bands at 3278, 2903, and 1580 cm^−1^, implying that compound **1** has phenolic group characteristics.

**Figure 2:**
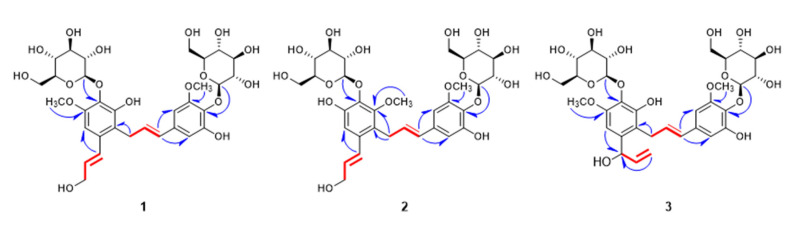
Key ^1^H-^1^H COSY (▬) and HMBC (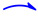) correlations of compounds **1**–**3**.

The ^1^H NMR spectroscopic data of **1** (Table 1, Figure S2) revealed one aromatic singlet signal (*δ*_H_ 6.71 (1H, H-6)) and 1,3,4,5-tetrasubstituted aromatic ring protons (*δ*_H_ 6.48 (1H, d, *J* = 2.0 Hz, H-2′) and 6.47 (1H, d, *J* = 2.0 Hz, H-6′)). The existence of two *trans*-olefinic groups was identified on the basis of the proton signals and coupling constants (*δ*_H_ 6.86 (1H, d, *J* = 15.5 Hz, H-7), 6.24 (1H, dt, *J* = 15.0, 5.0 Hz, H-8) and 6.20 (1H, dt, *J* = 15.0, 5.0 Hz, H-8′), 6.14 (1H, d, *J* = 15.5 Hz, H-7′)). Additionally, two methylene (*δ*_H_ 4.24 (2H, dd, *J* = 5.5, 2.0 Hz, H-9) and 3.55 (2H, d, *J* = 5.5 Hz, H-9′)) and two methoxy proton signals (*δ*_H_ 3.86 (3H, s, OCH_3_-5), 3.81 (3H, s, OCH_3_-5′)) were also observed in the ^1^H NMR. Two anomeric protons (*δ*_H_ 4.67 (1H, d, *J* = 8.0 Hz, Glc H-1), 4.63 (1H, d, *J* = 8.0 Hz, Glc H-1′)) and overlapped signals at *δ*_H_ 3.23–3.82 suggested that **1** has two sugars with *β* configuration [5]. The ^13^C NMR exhibited 32 signals, containing 12 signals for two glucopyranosyl moieties (Table 1, Figure S3). Enzyme hydrolysis of **1** and high performance liquid chromatography (HPLC) experiment led to the determination of the sugars of **1** as *β*-d-glucopyranose. In combination with ^13^C NMR and HMBC experimental results, compound **1** had two phenylpropanoid units that had π bond between *α* and *β* carbons (Figure S6). Each phenylpropyl moiety had one methoxy and one *β*-d-glucopyranose, approximate to demethyl syringin (**9**), supported by HMBC correlations (Figure 2). The differences were that one of the two phenylpropanoid units in **1** had 2-substitued aromatic ring and the other one had modified C-9′ into C-C bond instead of hydroxyl group. Furthermore, both of the substituted parts connected to each other were determined by HMBC correlation signal between H-9′ and C-2. Compound **1** was very similar to the known neolignan glucoside, tangshenoside III [6], except for missing one methoxy group in each of the two aromatic rings. Therefore, the structure of the new compound **1** was elucidated as 3,3′-demethyl tangshenoside III, and named as campanulalignan A.

Compound **2** was obtained as a pale yellow powder. The molecular formula of **2** was assigned as C_32_H_42_O_17_ by its HR-Q-TOF-MS ion peak at *m*/*z* 697.2354 [M−H]^−^ (calcd for C_32_H_41_O_17_, 697.2344) in negative mode (Figure S7) and was the same as **1**. The ^1^H and ^13^C NMR spectra of **2** strongly resembled those of **1**. However, the carbon chemical shift of one methoxy group in aromatic ring moved to the downfield (*δ*_C_ 62.7) in **2** (Table 1, Figure S9). In the ^1^H NMR, the difference between compounds **1** and **2** was shown on the aromatic singlet signal of **2** shifted to the downfield (*δ*_H_ 6.87 (1H, s, H-6)), whereas two olefinic protons of **2** shifted to the upfield (*δ*_H_ 6.81 (1H, d, *J* = 15.5 Hz, H-7) and *δ*_H_ 6.17 (1H, dt, *J* = 15.5, 6.0 Hz, H-8)) (Table 1, Figure S8). By analyzing the HMBC spectrum, the carbon that linked to the methoxy proton showed correlation with methylene proton peaks at *δ*_H_ 3.55 (2H, m, H-9′) (Figure 2). For these reasons, the location of one methoxy group was suggested to be C-3 of aromatic ring, rather than C-5 of **1**. Thus, the structure of the new compound **2** was elucidated as 5,3′-demethyl tangshenoside III, and named as campanulalignan B.

Compound **3** had a molecular formula of C_32_H_42_O_17_, as established by its molecular ion peak [M−H]^−^ at *m*/*z* 697.2336 (calcd for C_32_H_41_O_17_, 697.2344) in negative mode of HR-Q-TOF-MS (Figure S13) and was also the same as **1** and **2**. The ^1^H and ^13^C NMR spectra of **3** were almost identical with those of **1** and **2**, except for the existence of a vinyl alcohol group in **3** instead of an allyl alcohol group in **1** and **2**. ^1^H NMR spectra of **3** exhibited a vinyl alcohol signals at *δ*_H_ 5.40 (d, *J* = 5.5 Hz, H-7), 6.01 (ddd, *J* = 16.5, 10.0, 5.0 Hz, H-8), 5.26 (d, *J* = 17.0 Hz, H-9a), 5.12 (d, *J* = 10.5 Hz, H-9b) (Table 1). The ^13^C NMR and HSQC of **3** indicated the presence of a vinyl alcohol group, which includes one oxygenated carbon signal (*δ*_C_ 72.1 (C-7)), one olefinic carbon signal (*δ*_C_ 141.7 (C-8)), and one exomethylene signal (*δ*_C_ 115.2 (C-9)), (Table 1, Figure S16). The presence of the vinyl alcohol group in **3** was also supported by ^1^H-^1^H COSY data, which showed the correlation at H-7/H-8 and H-8/H-9a, 9b (Figure 2, Figure S17). The location of the vinyl alcohol group was unambiguously determined by the HMBC correlation from H-6 to C-7 to be at C-1. Two *β*-d-glucopyranosyl groups and two methoxy groups were located at C-4, C-4′, C-5, and C-5′, respectively, as **1**, through HMBC spectral interpretation (Figure 2). The chemical structure of **3** was established by the results noted above and by comparison with tangshenosides II and III [6,7], as shown in Figure 2, and named as campanulalignan C. However, the absolute configuration at C-7 of **3** was not determined due to the limited amount of sample obtained.

By comparison of their spectroscopic data with those published, the structures of the known compounds were identified as 3′,4-*O*-dimethylcedrusin (**4**) [8], 7*R*,8*S*-dihydrodehydrodiconiferyl alcohol (**5**) [9], 3′,4-*O*-dimethylcedrusin 9-*O*-*β*-glucopyranoside (**6**) [10], simplidin (**7**) [11], pinoresinol di-*O*-*β*-d-glucoside (**8**) [12], demethyl syringin (**9**) [13], ferulic acid (**10**) [14], 5-hydroxyconiferaldehyde (**11**) [15], icariside F_2_ (**12**) [16], benzyl-*α*-L-arabinopyranosyl-(1″ →6′)-*β*-d-glucopyranoside (**13**) [17], astragalin (**14**) [18], kaempferol 3-*O*-*β*-d-apiosyl(1→2)-*β*-d-glucopyranoside (**15**) [19], nicotiflorin (**16**) [20], kaempferol 3,7-di-*O*-*β*-d-glucoside (**17**) [21], and quercetin (**18**) [22].

### 2.2. Inhibitory Effects of Compounds on LPS-Induced PGE_2_ Production in RAW 264.7 Macrophages

The isolates **1**, **2,** and **4**–**18** were evaluated for their inhibitory effects of LPS-stimulated PGE_2_ production in RAW 264.7 macrophages. Compound **3** was not subjected to this experiment due to the limited amount obtained. Except for **2**, **3**, and **4**, all other compounds did not exhibit cytotoxicity in cell viability tests at concentrations up to 100 μM. The effects were assessed using the inhibitory rate on PGE_2_, which are listed in Table 2. Quercetin (**18**) showed the most potent inhibitory effect on LPS-induced PGE_2_ production in RAW 264.7 macrophages (97% inhibitory rate at 50 μM concentration), among isolated compounds from *C. takesimana*. 5-Hydroxyconiferaldehyde (**11**) also decreased remarkably the PGE_2_ production with 85.17% inhibitory rate. Additionally, 7*R*,8*S*-dihydrodehydrodiconiferyl alcohol (**5**), simplidin (**7**), pinoresinol di-*O*-*β*-d-glucoside (**8**), and ferulic acid (**10**) showed inhibitory effects over 50% on the PGE_2_ production at 50 μM concentration while the new compounds **1** and **2** did not exhibit a significant effect (<50% inhibitory rate). Among the active compounds, quercetin (**18**), 7*R*,8*S*-dihydrodehydrodiconiferyl alcohol (**5**), pinoresinol di-*O*-*β*-d-glucoside (**8**), and ferulic acid (**10**) have already been reported to have anti-inflammatory effects, whereas there is no report on biological activities related to anti-inflammatory effects for simplidin (**7**) and 5-hydroxyconiferaldehyde (**11**). Therefore, inhibitory effects of **7** and **11** on LPS-induced PGE_2_ production at various concentrations were investigated.

**Figure 3:**
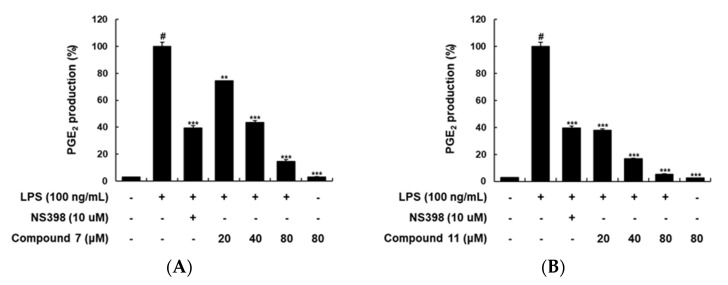
Inhibitory effects of compounds **7** (**A**) and **11** (**B**) on LPS-stimulated PGE_2_ production in RAW 264.7 macrophages. Cells were pretreated with different concentrations (20, 40, or 80 μM) of compounds **7** and **11** for 1 h, then with LPS (100 ng/mL), and incubated for 24 h. N-[2-(cyclohexyloxy)-4-nitrophenyl]-methanesulfonamide (NS-398, 10 μM) was used as a positive PGE_2_ production inhibitor. Level of PGE_2_ in culture media was quantified using enzyme-linked immunosorbent assay (ELISA) kits. (#: *p* < 0.05 as compared with the untreated group, ** *p* < 0.05, and *** *p* < 0.0002).

As shown in Figure 3A,B, simplidin (**7**) and 5-hydroxyconiferaldehyde (**11**) significantly and concentration-dependently suppressed LPS-stimulated PGE_2_ production. Compounds **7** and **11** did not influence the RAW 264.7 cell viability in either the presence or absence of LPS at concentrations up to 50 μM for 24 h, suggesting that **7** and **11** may be major constituents of the leaves of *C. takesimana* showing anti-inflammatory activities. Accordingly, further pharmacological studies are demanded to investigate the mechanisms responsible for the inhibitory effects of **7** and **11** on inflammatory mediator in RAW 264.7 macrophages.

## 3. Discussion

The investigation on secondary metabolites in the leaves of *C. takesimana* was carried out for the first time. Consequently, three new neolignan glucosides, campanulalignans A–C (**1**–**3**), were isolated by repeated chromatography. Additionally, five lignans (**4**–**8**), five phenolic compounds (**9**–**13**), and five flavonoids (**14**–**18**) were obtained from the present study. To the best of our knowledge, 3′,4-*O*-dimethylcedrusin 9-*O*-*β*-glucopyranoside (**6**), simplidin (**7**), 5-hydroxyconiferaldehyde (**11**), icariside F_2_ (**12**), benzyl-*α*-L-arabinopyranosyl-(1″→6′)-*β*-d-glucopyranoside (**13**), and kaempferol 3-*O*-*β*-d-apiosyl-(1→2)-*β*-d-glucopyranoside (**15**) were isolated from the Campanulaceae family for the first time in this work.

All the isolates from the leaves of *C. takesimana* except for compound **3** were estimated for their inhibitory effects on the production of PGE_2_ in the LPS-induced RAW 264.7 macrophages. Inflammatory mediators, such as NO and PGE_2_, and numerous pro-inflammatory cytokines released by the activated macrophages are important targets for the treatment of various inflammatory diseases, including multiple sclerosis, rheumatoid arthritis, and obesity [23,24]. iNOS and COX-2 protein expression are remarkably elevated in the inflammatory processes, resulting in increased production of NO and PGE_2_, respectively. These overexpressed pro-inflammatory mediators can lead to damage in living cells and tissues, resulting in various physiological disorders associated with inflammation [4,23]. Therefore, compounds that inhibit the production of pro-inflammatory mediators like NO and PGE_2_ can be candidates for anti-inflammatory drugs [4].

Among the isolates, well known quercetin (**18**) showed the strongest inhibitory effect [25]. In addition, 7*R*,8*S*-dihydrodehydrodiconiferyl alcohol (**5**), pinoresinol di-*O*-*β*-d-glucoside (**8**), and ferulic acid (**10**) exhibited remarkable inhibitory activity on LPS-stimulated PGE_2_ production in RAW264.7 cells and their inhibitory effects were well correlated with previous reports [26,27,28]. 7*R*,8*S*-Dihydrodehydrodiconiferyl alcohol (**5**), a neolignan, showed ABTS free radical scavenging and LPS-stimulated nitric oxide (NO) production inhibitory activities in a previous report [26]. Pinoresinol di-*O*-*β*-d-glucoside (**8**) possesses protective activity on human endothelial cells by inhibiting oxidized low density lipoprotein (oxLDL)-induced SOD activity, NO production, and endothelial nitric oxide synthase (eNOS) expression [27]. A prior study revealed that ferulic acid (**10**) significantly reduced LPS-induced PGE_2_ levels [28].

Simplidin (**7**) and 5-hydroxyconiferaldehyde (**11**) also showed significant inhibitory effects on PGE_2_ production in this study, but there was no investigation on biological activities related to anti-inflammatory effects in previous studies. Simplidin (**7**), first isolated from *Firmiana simplex* (Sterculiaceae) [11], exhibited cytotoxicities against human cancer cell lines [29]. 5-Hydroxyconiferaldehyde (**11**) was discovered in the biosynthetic pathway for syringyl monolignol in angiosperms [30] and identified from the methanol extract of *Carthamus tinctorius* seed [15]. However, to the best of our knowledge, there have been no reports of any biological or pharmacological activities for 5-hydroxyconiferaldehyde (**11**). In our investigation and for the first time, simplidin (**7**) and 5-hydroxyconiferaldehyde (**11**) are suggested as active natural products with inhibitory effects on a pro-inflammatory mediator, PGE_2_ production.

## 4. Materials and Methods

### 4.1. Plant Material

The leaves of *Campanula takesimana* Nakai (Campanulaceae; Korean bellflower) used in present study were provided by COSMAX BIO, Gyeonggi-do, Republic of Korea, in July 2019. The origin of the material was identified by D.S.J., one of authors, and a voucher specimen (CATA-2019) has been deposited in the Laboratory of Natural Product Medicine, College of Pharmacy, Kyung Hee University, Seoul, Republic of Korea.

### 4.2. General Experimental Procedures

General experimental procedures are in the Supplementary Materials.

### 4.3. Extraction and Isolation

Dried leaves of *C. takesimana* (2.5 kg) were extracted with distilled water (60 L) at 100 °C for 5 h, and the solvent was evaporated by rotary evaporator at 45 °C. The hot water extract (1.0 kg) was separated over column chromatography (CC) using Diaion HP-20 as the stationary phase with a gradient system of acetone-H_2_O (0:100 to 100:0, *v*/*v*) to give 17 fractions (F1~F17).

Fraction F10 was fractionated further using Sephadex LH-20 CC (5.5 × 45.9 cm) with 40% acetone to produce eight fractions (F10-1~F10-8). Fraction F10-4 was subjected to silica gel CC (230–400 mesh; 5.1 × 33.0 cm, CH_2_Cl_2_-MeOH-H_2_O = 80:18:2 to 70:27:3, *v*/*v*/*v*), yielding **9** (78.5 mg, yield 0.00314% by dry weight of the plant material). Fraction F10-5 was separated through silica gel CC (230–400 mesh; 5.1 × 33.0 cm, CH_2_Cl_2_-MeOH-H_2_O = 80:18:2 to 70:27:3, *v*/*v*/*v*) to get **12** (3.7 mg, 0.000148%) and **13** (3.0 mg, 0.00012%). Fraction F12 was subjected to Sephadex LH-20 CC (4.5 _×_ 45.9 cm) with MeOH-H_2_O mixture (70:30 *v*/*v*) to give 10 subfractions (F12-1~F12-10). Subfraction F12-2 was fractionated further by using silica gel CC (230–400 mesh; 3.1 × 25.0 cm, CH_2_Cl_2_-MeOH-H_2_O = 85:13.5:1.5 to 70:27:3, *v*/*v*/*v*) to produce 14 subfractions (F12-2-1~F12-2-14). Compound **6** (175.3 mg, 0.00701%) was isolated from subfraction F12-2-6 by a flash chromatographic system with a Redi Sep-C18 cartridge (43 g, MeOH-H_2_O, from 15:85 to 45:55, *v*/*v*). Subfraction F12-2-9 was also subjected to a flash chromatographic system by a Redi Sep-C18 cartridge (43 g, MeOH-H_2_O, from 13:87 to 40:60, *v*/*v*) to afford **8** (14.3 mg, 0.000572%). Subfraction F12-2-10 was fractioned by a flash chromatographic system with a Redi Sep-C18 cartridge (43 g, MeOH-H_2_O, 27:72 to 50:50, *v*/*v*) to isolate **1** (20.2 mg, 0.000808%), **2** (8.0 mg, 0.00032%), and **17** (8.7 mg, 0.000348%). Fraction F13 was separated into six subfractions (F13-1~F13–6) using Sephadex LH-20 CC (5.5 × 47.9 cm) with 50% acetone. Compounds **1** (3.0 mg, 0.00012%) and **3** (2.5 mg, 0.0001%) were purified from subfraction F13-2 using a preparative HPLC with Gemini 5 μm NX-C18 110A column (acetonitirle-H_2_O = 15:85 to 30:70, *v*/*v*). Fraction F15 was subjected to Sephadex LH-20 CC (5.6 × 58.6 cm) with 60% acetone, and produced six fractions (F15-1~F15-6). Fraction F15-2 was fractionated using silica gel CC (230–400 mesh; 5.1 × 33.0 cm, CH_2_Cl_2_-MeOH-H_2_O = 90:9:1 to 60:36:4, *v*/*v*/*v*), and produced 16 subfractions (F15-2-1~F15-2-16). Compounds **6** (28.0 mg, 0.00112%) and **7** (7.0 mg, 0.00028%) were obtained from subfraction F15-2 by a flash chromatographic system with a Redi Sep-C18 cartridge (30 g, MeOH-H_2_O = 20:80 to 40:60, *v*/*v*). Compound **11** (3.7 mg, 0.000148%) was purified from subfraction F15-5-2 by silica gel CC (230–400 mesh; 2.5 × 20.0 cm, CH_2_Cl_2_-MeOH-H_2_O = 95:4.5:0.5 to 60:36:4 *v*/*v*/*v*). Fraction F15-5 was subjected to silica gel CC (230–400 mesh; 5.1 × 33.0 cm, CH_2_Cl_2_-MeOH-H_2_O = 90:9:1 to 60:36:4, *v*/*v*/*v*) to isolate **10** (11.2 mg, 0.000448%), **14** (91.2 mg, 0.00365%), **15** (17.0 mg, 0.000704%), and **16** (32.4 mg, 0.0013%). Fraction F16 was separated into nine subfractions (F16-1~F16-9) using Sephadex LH-20 CC (4.5 × 45.9 cm) with 70% MeOH. Subfraction F16-1 was subjected to silica gel CC (230–400 mesh; 5.1 × 33.0 cm, CH_2_Cl_2_-MeOH-H_2_O = 90:9:1 to 60:36:4, *v*/*v*/*v*) to afford **4** (293.0 mg, 0.0117%) and **5** (79.5 mg, 0.00318%). Compound **18** (10.1 mg, 0.000404%) was purified by recrystallizing subfraction F17 with MeOH.

#### 4.3.1. Campanulalignan A (**1**)

Pale yellow powder; HR-Q-TOF-MS (negative mode) *m*/*z* = 697.2360 [M–H]^−^ (calcd for C_32_H_41_O_17_, 693.2344); ${\left[\alpha \right]}_{D}^{20}$: −12.7° (*c* 0.1, MeOH); UV (MeOH) λ_max_ nm (log ε): 224 (3.31), 265 (3.08); IR (ATR) ν_max_ 3278, 2903, 1580, 1448, 1348 cm^−1^; ^1^H and ^13^C NMR data, see Table 1.

#### 4.3.2. Campanulalignan B (**2**)

Pale yellow powder; HR-Q-TOF-MS (negative mode) *m*/*z* = 697.2354 [M–H]^−^ (calcd for C_32_H_41_O_17_, 693.2344); ${\left[\alpha \right]}_{D}^{20}$: −14.5° (*c* 0.1, MeOH); UV (MeOH) λ_max_ nm (log ε): 222 (3.24), 263 (3.00); IR (ATR) ν_max_ 3302, 2920, 1580, 1421, 1347 cm^−1^; ^1^H and ^13^C NMR data, see Table 1.

#### 4.3.3. Campanulalignan C (**3**)

Brown amorphous powder; HR-Q-TOF-MS (negative mode) *m*/*z* = 697.2336 [M–H]^−^ (calcd for C_32_H_41_O_17_, 693.2344); ${\left[\alpha \right]}_{D}^{20}$: −24.5° (*c* 0.1, MeOH); UV (MeOH) λ_max_ nm (log ε): 230 (3.47), 267 (3.22); IR (ATR) ν_max_ 3266, 2922, 1585, 1342, 1017 cm^−1^; ^1^H and ^13^C NMR data, see Table 1.

### 4.4. Enzymatic Hydrolysis of Compound **1**

Compound **1** (1.0 mg) was incubated together with 2 drops of toluene, *β*-glucosidase (2.5 mg), and H_2_O (1.5 mL) in a CO_2_ incubator at 35 °C for 3 days. The reaction was stopped by addition EtOH to the reaction mixture and *β*-glucosidase was removed by filtration. The presence of glucose in **1** was confirmed by co-TLC (R*_f_* 0.3, *n*-BuOH:acetic acid:H_2_O = 2:1:1, *v*/*v*/*v*) with a standard compound.

### 4.5. Absolute Configurations of β-glucose in Compound **1**

The absolute configuration of *β*-glucose in **1** was confirmed by the method from Tanaka et al. [31]. Hydrolysate was dissolved in pyridine (500 μL) and l-cysteine methyl ester hydrochloride (1.2 mg) was added and heated at 60 °C for 1 h. The mixture was heated again at 60 °C for 1 h after adding *σ*-tolyl isothiocyanate (100 μL) and analyzed directly by HPLC under a gradient system (A: 0.1% (*v*/*v*) formic acid in water, B: 0.1% (*v*/*v*) formic acid in acetonitrile, 10 to 50% B, 45 min). The reaction mixture of **1** was detected at 27.0 min. The retention times of authentic l- and d-glucoses were 26.1 and 27.0 min, respectively, under the same HPLC conditions. Thus, the absolute configuration of *β*-glucose in **1** was confirmed as the D configuration.

### 4.6. Cell Culture

RAW 264.7 macrophages were purchased from the Korea Cell Line Bank (Seoul, South Korea). Cells were incubated in Dulbecco’s modified Eagle’s medium (DMEM) supplemented with penicillin–streptomycin sulfate (100 units/mL and 100 μg/mL) and 10% FBS at 37 °C humidified incubator containing of 5% CO_2_.

### 4.7. Cell Viability Test

Cell viability test was assessed by the MTT (3-[4,5-dimethylthiazol-2-yl]-2,5-dipheyl tetrazoliumbromide; Sigma-Aldrich) assay. RAW 264.7 macrophages were grown with each isolated compound and LPS (100 ng/mL) for 24 h. After 24 h, cells were treated with MTT solution for 4 h. MTT formazan was resolved by adding dimethyl sulfoxide (DMSO), and the absorbance of each well at 540 nm was read by a microplate reader.

### 4.8. Measurement of PGE_2_ Production

RAW 264.7 cells were treated with compounds (50 μM) 1 h prior to LPS (100 ng/mL) stimulation for 24 h. Cells were pretreated with positive controls (NS398) for 1 h, and then stimulated with LPS (100 ng/mL) for 24 h. The supernatant was collected and PGE_2_ production detected by using PGE_2_ ELISA Kits (R&D Systems, MN, USA).

## 5. Conclusions

Three new neolignan glucosides (**1**–**3**) and 15 known compounds were isolated from the leaves of *C. takesimana* in the present study. We found that 7*R*,8*S*-dihydrodehydrodiconiferyl alcohol (**5**), simplidin (**7**), pinoresinol di-*O*-*β*-d-glucoside (**8**), ferulic acid (**10**), 5-hydroxyconiferaldehyde (**11**), and quercetin (**18**) inhibit production of an inflammatory mediator PGE_2_. Among these, the inhibitory effect of simplidin (**7**) and 5-hydroxyconiferaldehyde (**11**) on PGE_2_ production was reported for the first time in this work. Thus, simplidin (**7**) and 5-hydroxyconiferaldehyde (**11**) are worthy of further pharmacological evaluation for their potential as anti-inflammatory drugs.

## Figures and Tables

**Table 1:** ^1^H and ^13^C NMR spectroscopic data of compounds **1**–**3** (*δ* in ppm, CD_3_OD, 500 and 125 MHz).

Position *^a^*	1		2		3	
	*δ*_C_	*δ*_H_ *^b^*	*δ*_C_	*δ*_H_ *^b^*	*δ*_C_	*δ*_H_ *^b^*
1	134.9		135.4		140.0	
2	119.5		123.9		119.0	
3	152.4		153.0		150.1	
4	134.9		139.3		134.6	
5	150.1		150.8		152.5	
6	102.5	6.71 s	110.8	6.87 s	102.9	6.71 s
7	129.3	6.86 d (15.5)	128.9	6.81 d (15.5)	72.1	5.40 d (5.5)
8	132.0	6.24 dt (15.0, 5.0)	130.0	6.17 dt (15.5, 6.0)	141.7	6.01 ddd (16.5, 10.0, 5.0)
9	62.2	4.24 dd (5.5, 2.0)	63.9	4.21 dd (5.5, 2.0)	115.2	5.26 d (17.0)
						5.12 d (10.5)
1′	136.9		136.6		136.9	
2′	108.2	6.48 d (2.0)	108.2	6.49 d (2.0)	103.1	6.47 d (1.5)
3′	151.9		152.0		152.5	
4′	134.5		134.6		134.6	
5′	154.1		154.3		154.3	
6′	103.0	6.47 d (2.0)	103.1	6.48 d (2.0)	108.4	6.48 d (1.5)
7′	130.9	6.14 (15.5)	130.4	6.13 d (15.5)	131.0	6.18 d (16.0)
8′	129.9	6.20 dt (15.0, 5.0)	131.4	6.24 dt (15.5, 5.5)	130.1	6.24 dt (15.5, 6.0)
9′	29.9	3.55 d (5.5)	30.2	3.55 d (5.5)	29.5	3.62 d (7.0)
						3.54 d (6.0)
Glc-1	107.3	4.67 d (8.0)	107.3	4.81 d (8.0)	107.3	4.67 d (8.0)
Glc-2	71.0	3.45 overlap	71.0	3.49 m	71.0	3.51 overlap
Glc-3	77.8	3.40 overlap	78.0	3.43 overlap	77.8	3.45 overlap
Glc-4	75.5	3.49 overlap	75.5	3.43 overlap	75.5	3.42 overlap
Glc-5	78.5	3.22 m	78.6	3.22 overlap	78.7	3.23 overlap
Glc-6	62.0	3.68/3.82 overlap	62.4	3.82 overlap	62.3	3.86 m
Glc-1′	107.0	4.63 d (8.0)	107.0	4.64 d (8.0)	107.1	4.63 d (7.5)
Glc-2′	71.0	3.47 overlap	71.0	3.47 m	71.0	3.51 overlap
Glc-3′	77.8	3.43 overlap	77.9	3.84 overlap	77.8	3.45 overlap
Glc-4′	75.5	3.51 m	75.5	3.44 m	75.5	3.42 overlap
Glc-5′	78.5	3.30 m	78.5	3.29 m	78.7	3.23 overlap
Glc-6′	62.0	3.68/3.82 overlap	62.2	3.22 overlap	62.3	3.78 m
OCH_3_-5	56.8	3.86 s	62.7	3.87s	56.8	3.84 s
OCH_3_-5′	56.8	3.81 s	56.8	3.81s	56.7	3.81 s

***^a^*** All assignments were based on ^1^H-^1^H correlation spectroscopy (COSY), ^1^H-^13^C heteronuclear single quantum coherence spectroscopy (HSQC), and ^1^H-^13^C heteronuclear multiple bond correlation (HMBC) results. ***^b^*** *δ*_H_ (multi *J* in Hz).

**Table 2:** The cytotoxicities and inhibitory effects of the isolates from *C. takesimana* on lipopolysaccharide (LPS)-induced prostaglandin E_2_ (PGE_2_) production in RAW 264.7 macrophages.

Compound	IC_80_ (μM) Cytotoxicitiy	Inhibition Rate (%) *^a^*	Compound	IC_80_ (μM) Cytotoxicitiy	Inhibition Rate (%) *^a^*
**1**	>50	23.92	**10**	>50	62.53
**2**	33.66	33.07	**11**	>50	85.17
**3**	-	-	**12**	>50	31.07
**4**	30.69	7.89	**13**	>50	33.12
**5**	>50	69.35	**14**	>50	23.55
**6**	>50	20.01	**15**	>50	43.49
**7**	>50	60.01	**16**	>50	36.73
**8**	>50	58.08	**17**	>50	34.51
**9**	>50	37.44	**18**	>50	97.01

***^a^*** Cells were pretreated with 50 μM concentration of compounds for 1 h, then with LPS (100 ng/mL), and incubated for 24 h.

## Supplementary Materials

The following are available online at https://www.mdpi.com/2223-7747/9/9/1232/s1, Figure S1: HR-Q-TOF-MS spectrum of compound **1**, Figure S2: The ^1^H-NMR (500 MHz, CD_3_OD) spectrum of compound **1**, Figure S3: The ^13^C-NMR (125 MHz, CD_3_OD) spectrum of compound **1**, Figure S4: The HSQC spectrum of compound **1** in CD_3_OD, Figure S5: The COSY spectrum of compound **1** in CD_3_OD, Figure S6: The HMBC spectrum of compound **1** in CD_3_OD, Figure S7: HR-Q-TOF-MS spectrum of compound **2**, Figure S8: The ^1^H-NMR (500 MHz, CD_3_OD) spectrum of compound **2**, Figure S9: The ^13^C-NMR (125 MHz, CD_3_OD) spectrum of compound **2**, Figure S10: The HSQC spectrum of compound **2** in CD_3_OD, Figure S11: The COSY spectrum of compound **2** in CD_3_OD, Figure S12: The HMBC spectrum of compound **2** in CD_3_OD, Figure S13: HR-Q-TOF-MS spectrum of compound **3**, Figure S14: The ^1^H-NMR (500 MHz, CD_3_OD) spectrum of compound **3**, Figure S15: The ^13^C-NMR (125 MHz, CD_3_OD) spectrum of compound **3**, Figure S16: The HSQC spectrum of compound **3** in CD_3_OD, Figure S17: The COSY spectrum of compound **3** in CD_3_OD, Figure S18: The HMBC spectrum of compound **3** in CD_3_OD
