# Supplementary material for: Chemical Constituents of the Leaves of Campanula takesimana (Korean Bellflower) and Their Inhibitory Effects on LPS-induced PGE2 Production
Source: Plants (Basel). 2020 Sep 18;9(9):1232. doi: 10.3390/plants9091232 (PMC7570004; doi:10.3390/plants9091232)

plants

Supplementary materials

Chemical Constituents of the Leaves of *Campanula takesimana* (Korean Bellflower) and Their Inhibitory Effects on LPS-induced PGE_2 Production

Contents

General Experimental procedure
Figure S1. HR-Q-TOF-MS spectrum of compound **1**
Figure S2. The ^{1}H-NMR (500 MHz, CD_3OD) spectrum of compound **1**
Figure S3. The ^{13}C-NMR (125 MHz, CD_3OD) spectrum of compound **1**
Figure S4. The HSQC spectrum of compound **1** in CD_3OD
Figure S5. The COSY spectrum of compound **1** in CD_3OD
Figure S6. The HMBC spectrum of compound **1** in CD_3OD
Figure S7. HR-Q-TOF-MS spectrum of compound **2**
Figure S8. The ^{1}H-NMR (500 MHz, CD_3OD) spectrum of compound **2**
Figure S9. The ^{13}C-NMR (125 MHz, CD_3OD) spectrum of compound **2**
Figure S10. The HSQC spectrum of compound **2** in CD_3OD
Figure S11. The COSY spectrum of compound **2** in CD_3OD
Figure S12. The HMBC spectrum of compound **2** in CD_3OD
Figure S13. HR-Q-TOF-MS spectrum of compound **3**
Figure S14. The ^{1}H-NMR (500 MHz, CD_3OD) spectrum of compound **3**
Figure S15. The ^{13}C-NMR (125 MHz, CD_3OD) spectrum of compound **3**
Figure S16. The HSQC spectrum of compound **3** in CD_3OD
Figure S17. The COSY spectrum of compound **3** in CD_3OD
Figure S18. The HMBC spectrum of compound **3** in CD_3OD

General Experimental procedure

UV spectra were evaluated on Optizen pop (Mecasys, Daejeon, Korea). MPA 100 (Stanford research systems, Sunnyvale, CA, USA) was used to measure melting points in open capillary tubes. Optical rotations were obtained on a Jasco P-2000 polarimeter (JASCO, Tokyo, Janpan), using a 10-cm microcell. JEOL (JEOL, Tokyo, Japan) 500 MHz was used for obtaining NMR spectra. HR-Mass spectra were obtained by a Q-TOF micro mass spectrometer (Waters, Milford, Massachusetts, USA). TLC analyses were performed on Silica gel 60 F_{254} (Merck, Kenilworth, MA, USA) and RP-18 F_{254S} (Merck) plates. Compounds were visualized by dipping plates into 20% (v/v) H_2SO_4 reagent (Samchun) and then heated at 110°C for 5-10 min. Agilent Cary 630 FTIR (Agilent Technologies, Santa Clara, CA, USA) was applied to obtain IR spectrum. Sephadex LH-20 (Amersham Pharmacia Biotech, Buckinghamshire, United Kingdom), Silica gel (Merck 60A, 230-400 mesh ASTM), Diaion HP-20 (Mitsubishi, Tokyo, Japan), and reversed-phase silica gel (YMC Co., ODS-A 12 nm S-150 μm) were used for column chromatography. Pre-packed cartridges, Redi Sep-Silica (12 g, 24 g, 40 g, Teledyne Isco) and Redi Sep-C18 (13 g, 26 g, 43 g, 130 g, Teledyne Isco) were used for flash chromatography. HPLC was performed using Waters purification system (1525 pump, PDA 1996 detector) with Gemini NX-C18 110A column (250 × 21.2mm i.d. 5 μm, Phenomenex, Torrance, CA, USA). Flash chromatography was performed using the flash purification system (Combi Flash Rf, Teledyne Isco). Before chromatographic separations, all solvents used for this study were distilled.

Figure S1. HR-Q-TOF-MS spectrum of compound **1**

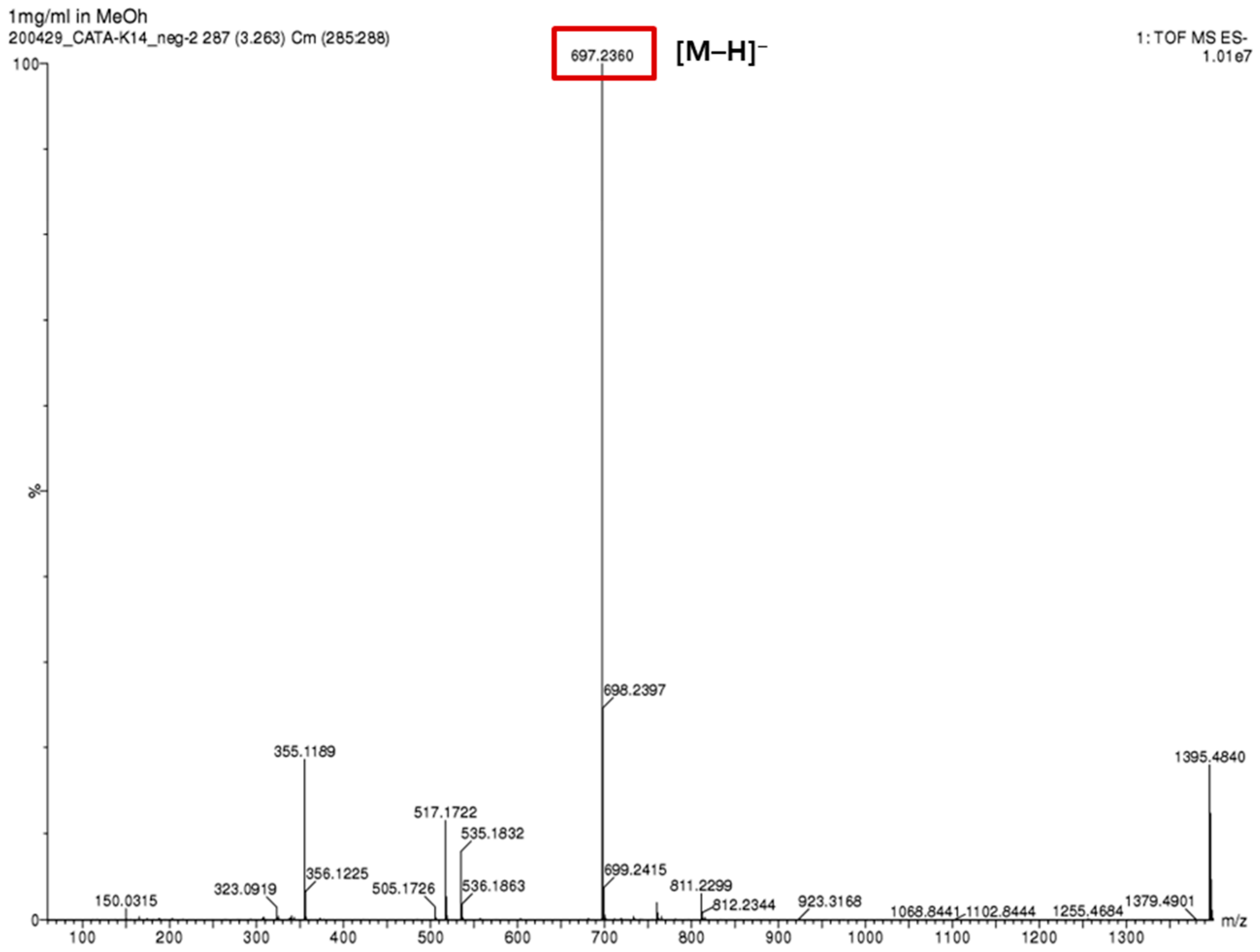

Figure S2. The ^{1}H-NMR (500 MHz, CD_3OD) spectrum of compound **1**

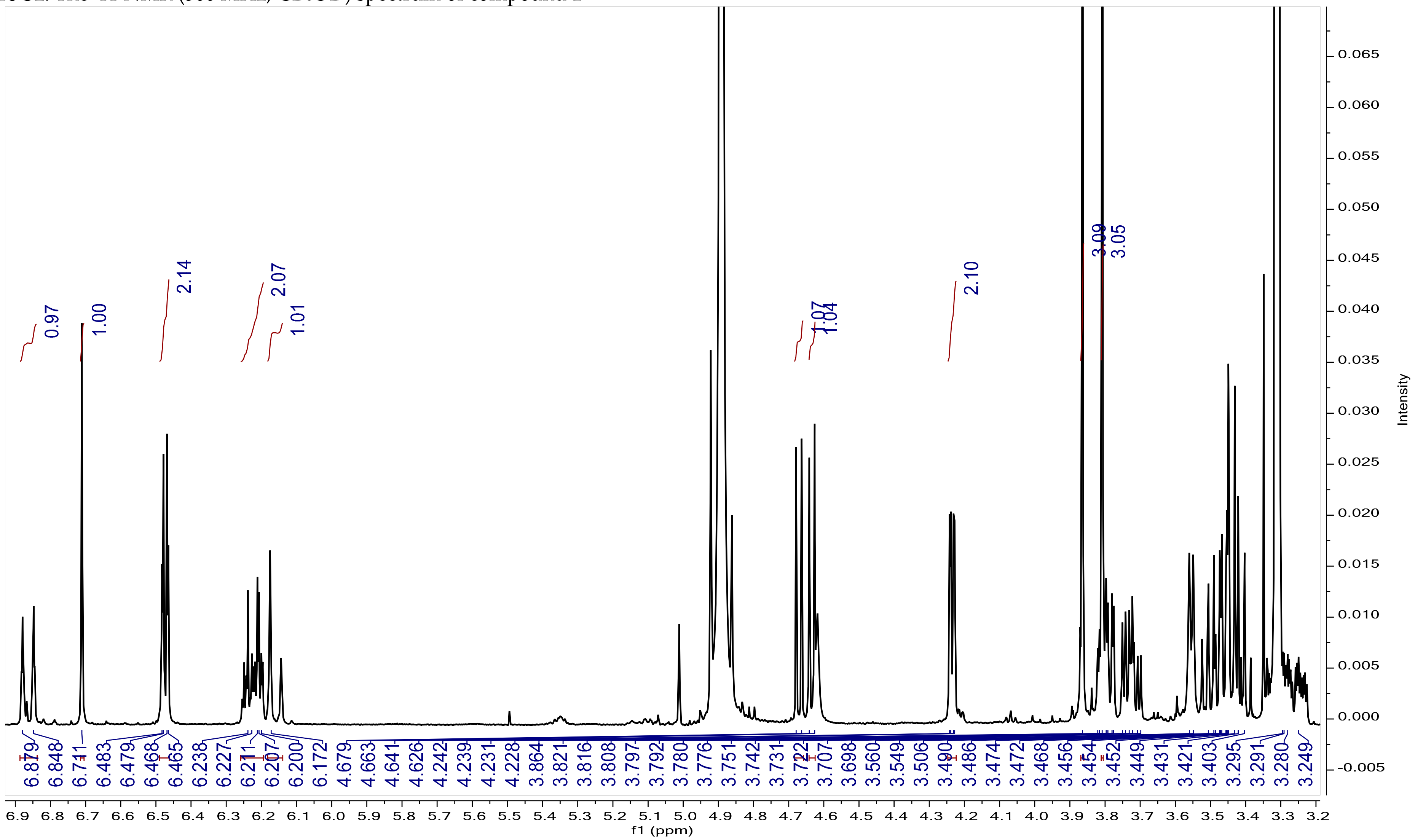

Figure S3. The ^{13}C-NMR (125 MHz, CD_3OD) spectrum of compound **1**

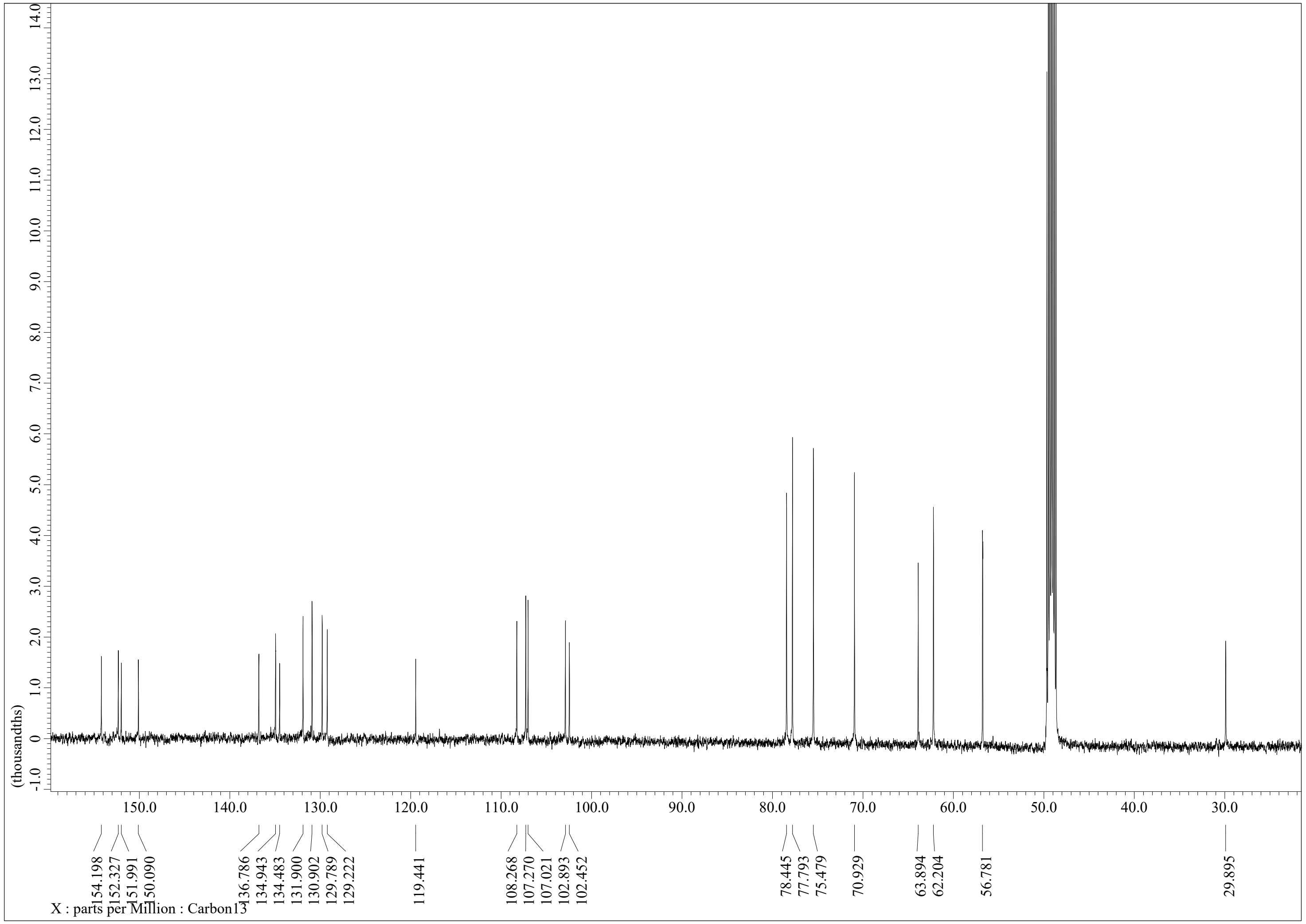

Figure S4. The HSQC spectrum of compound **1** in CD_3OD

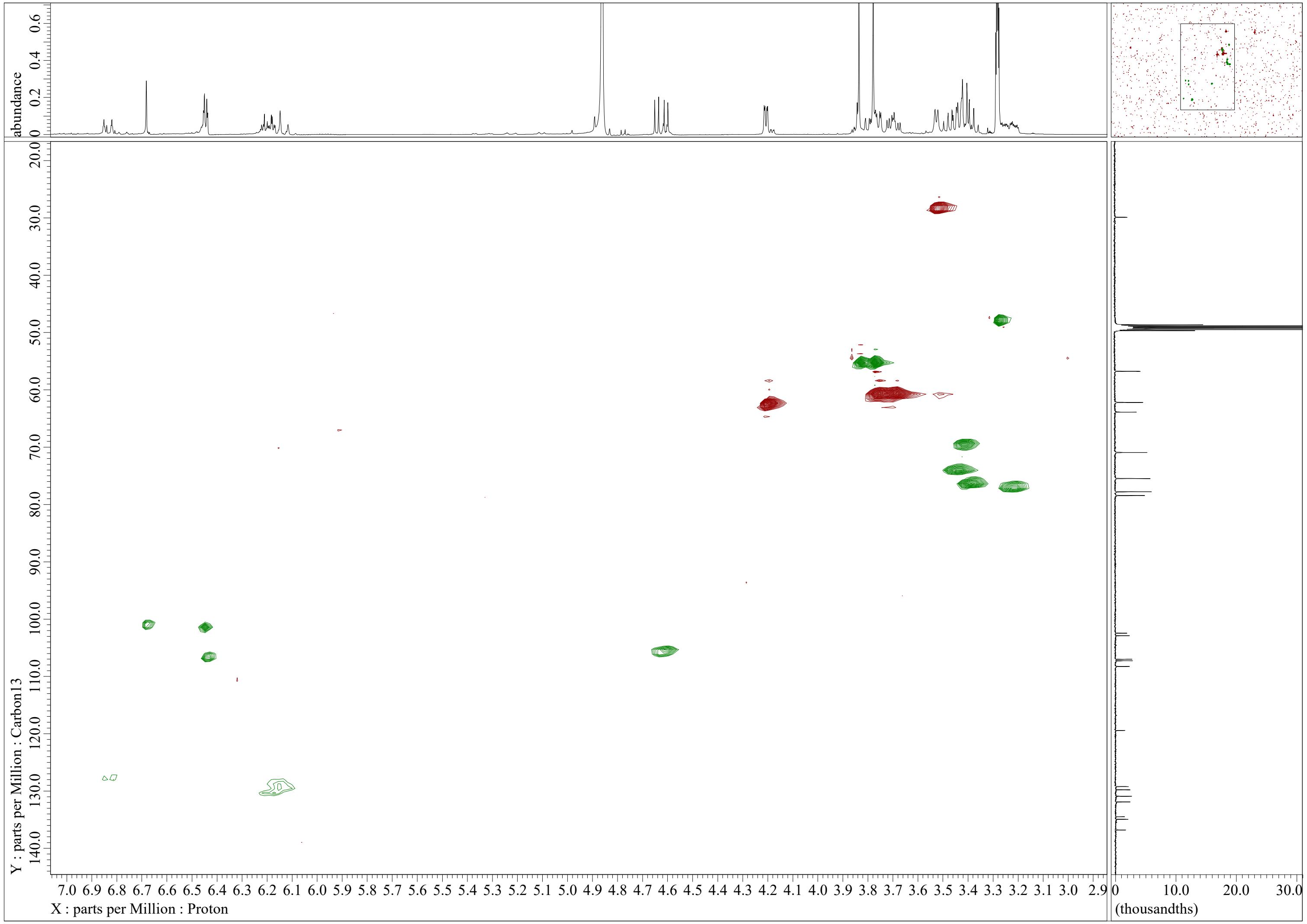

Figure S5. The COSY spectrum of compound **1** in CD_3OD

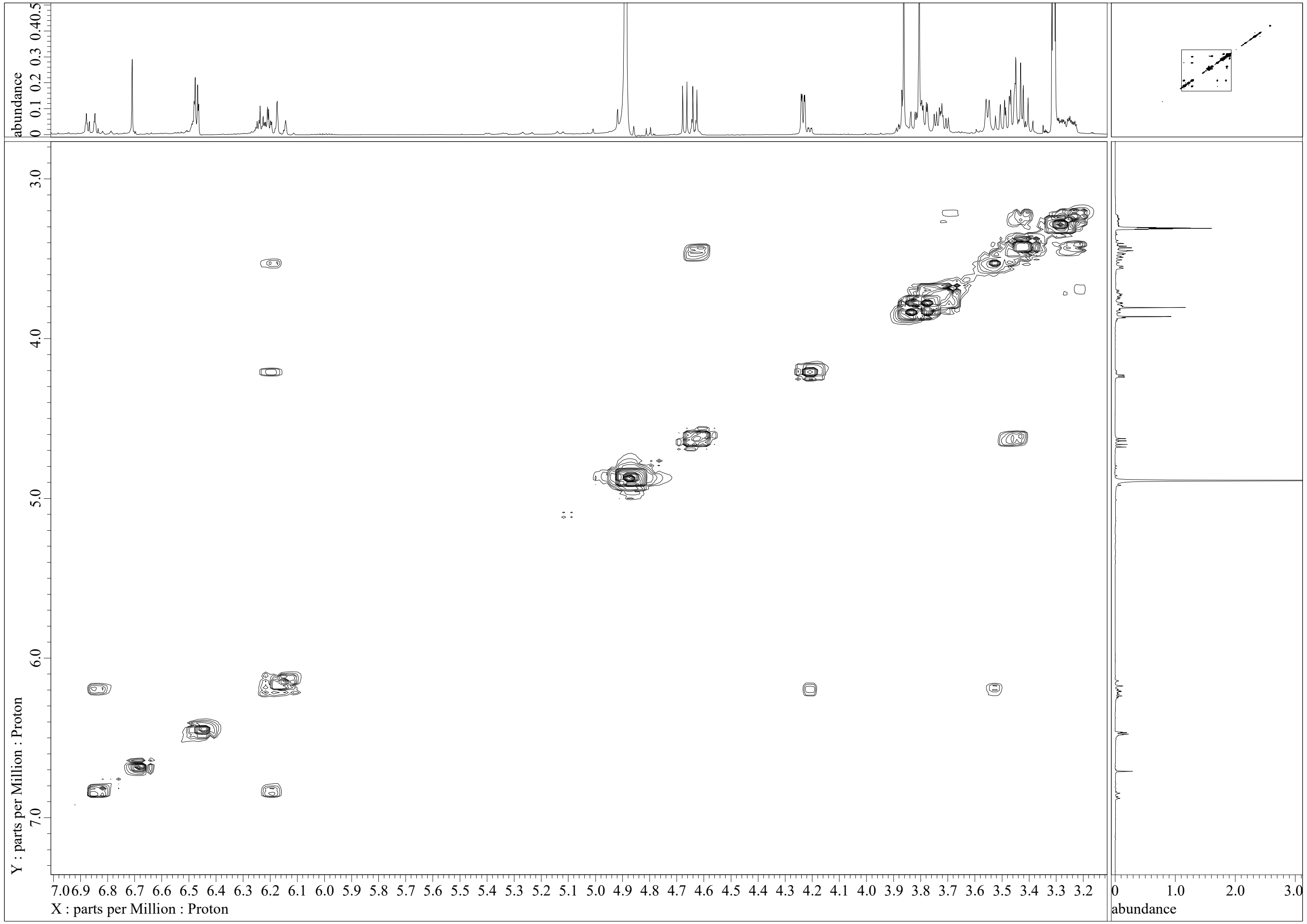

Figure S6. The HMBC spectrum of compound **1** in CD3OD

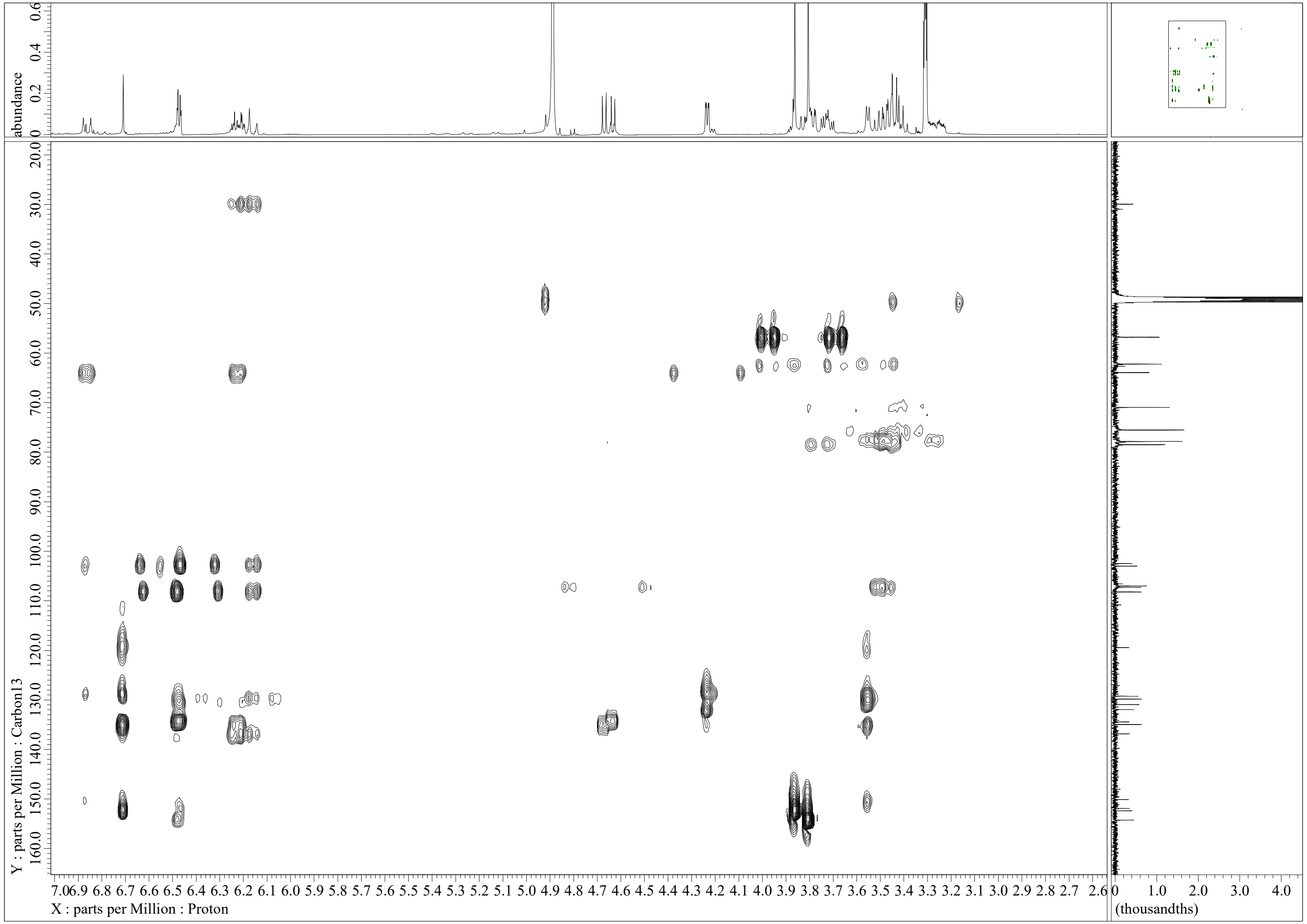

Figure S7. HR-Q-TOF-MS spectrum of compound **2**

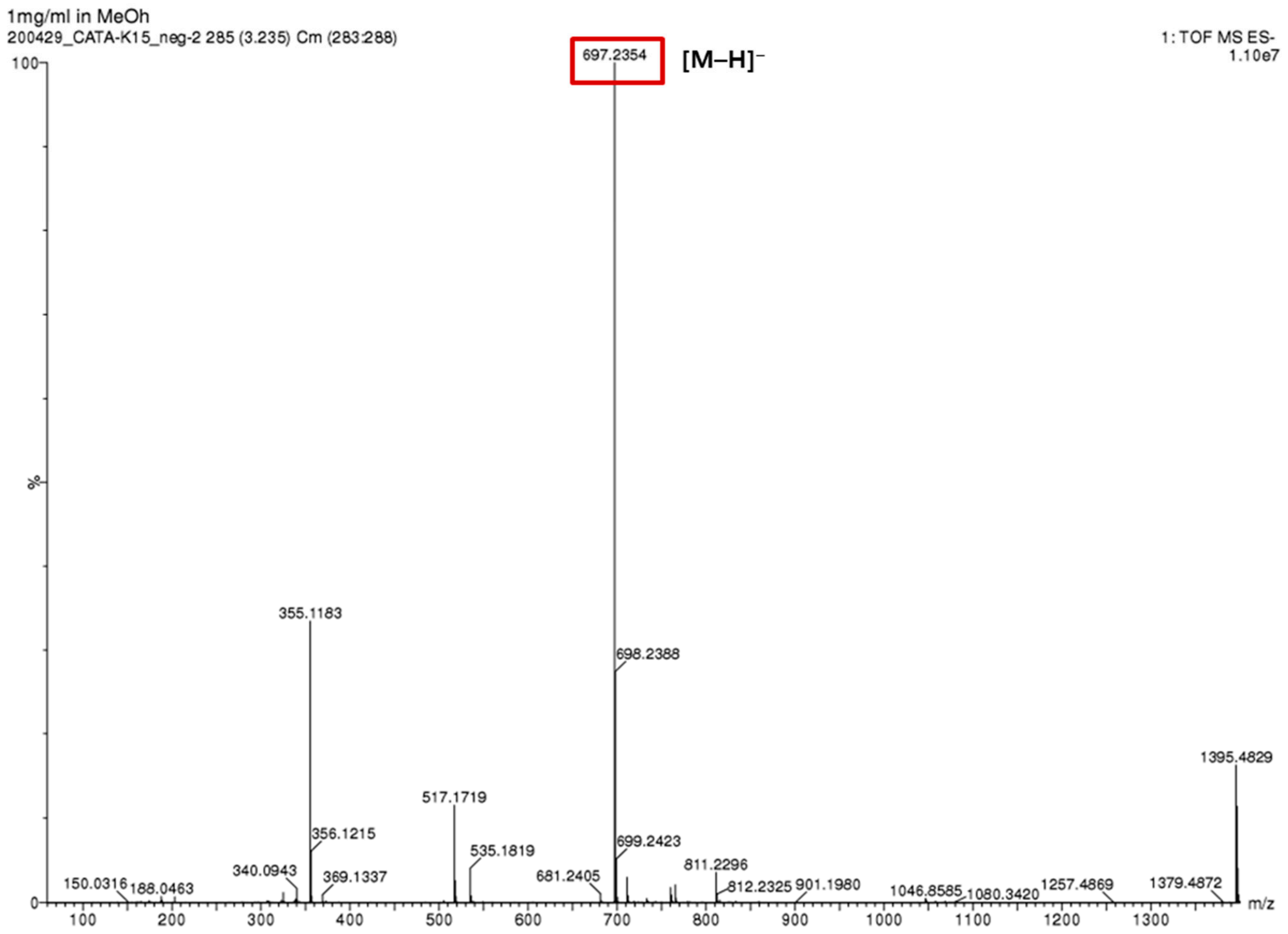

Figure S8. The ^{1}H-NMR (500 MHz, CD_3OD) spectrum of compound **2**

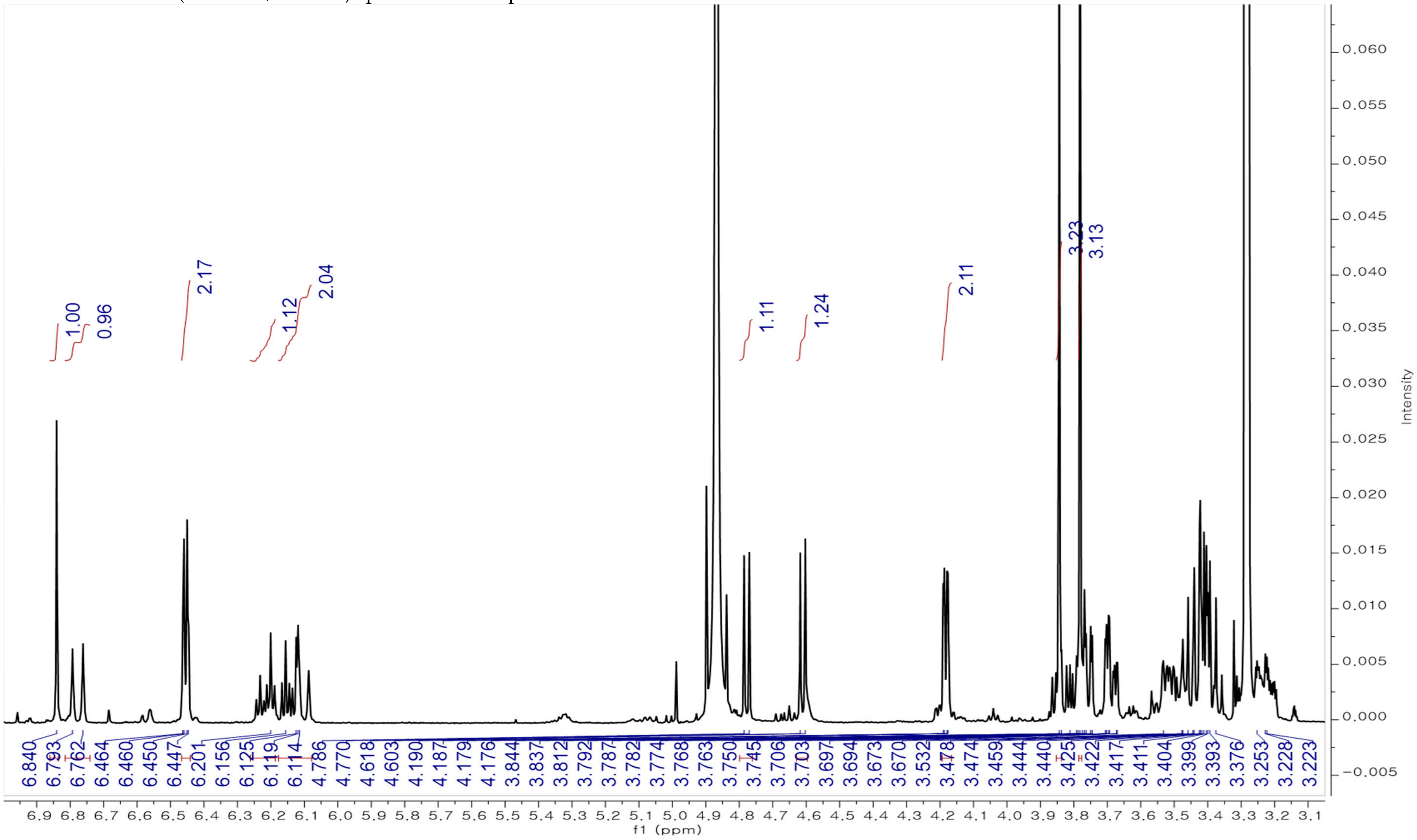

Figure S9. The ^{13}C-NMR (125 MHz, CD_3OD) spectrum of compound **2**

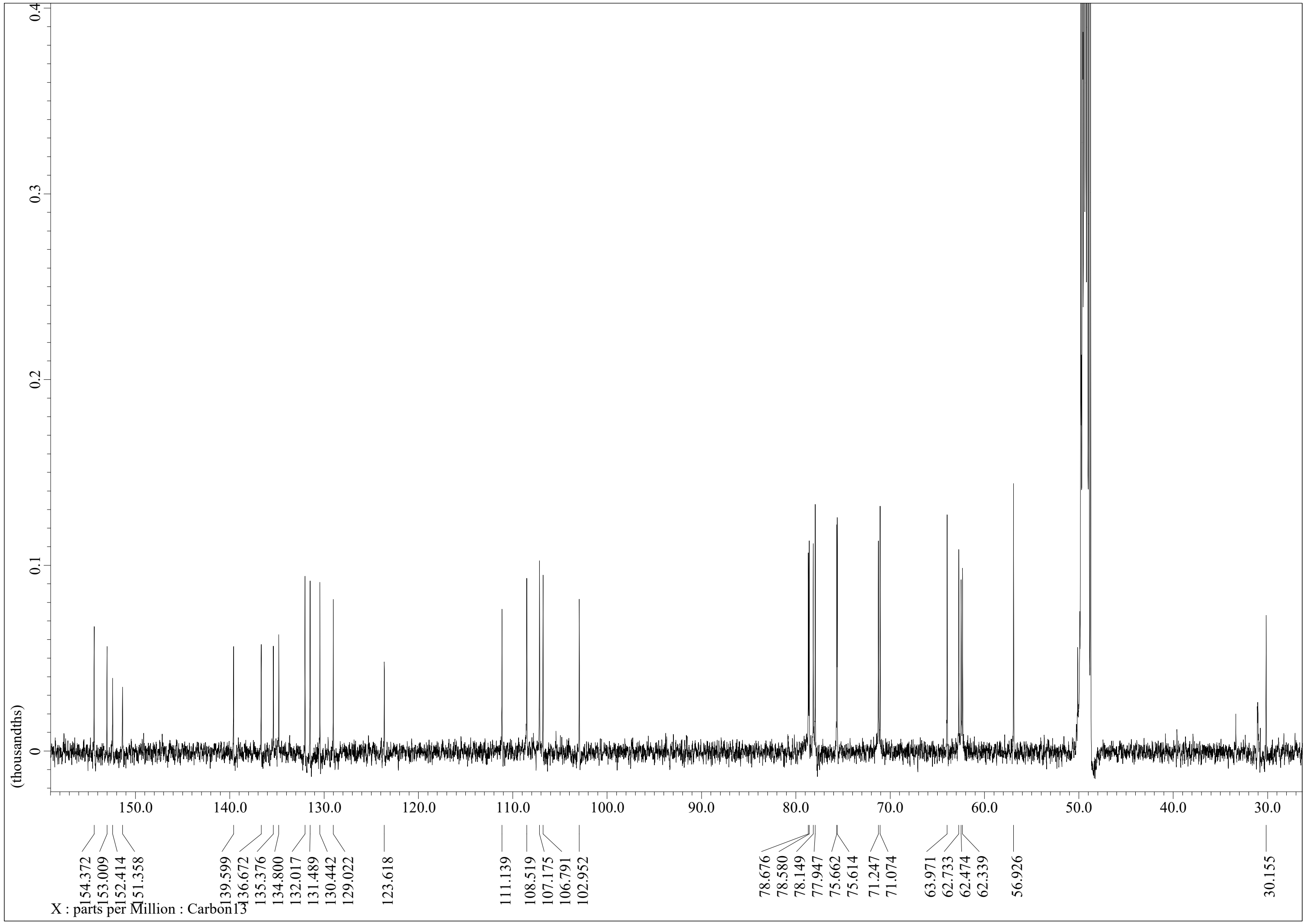

Figure S10. The HSQC spectrum of compound **2** in CD_3OD

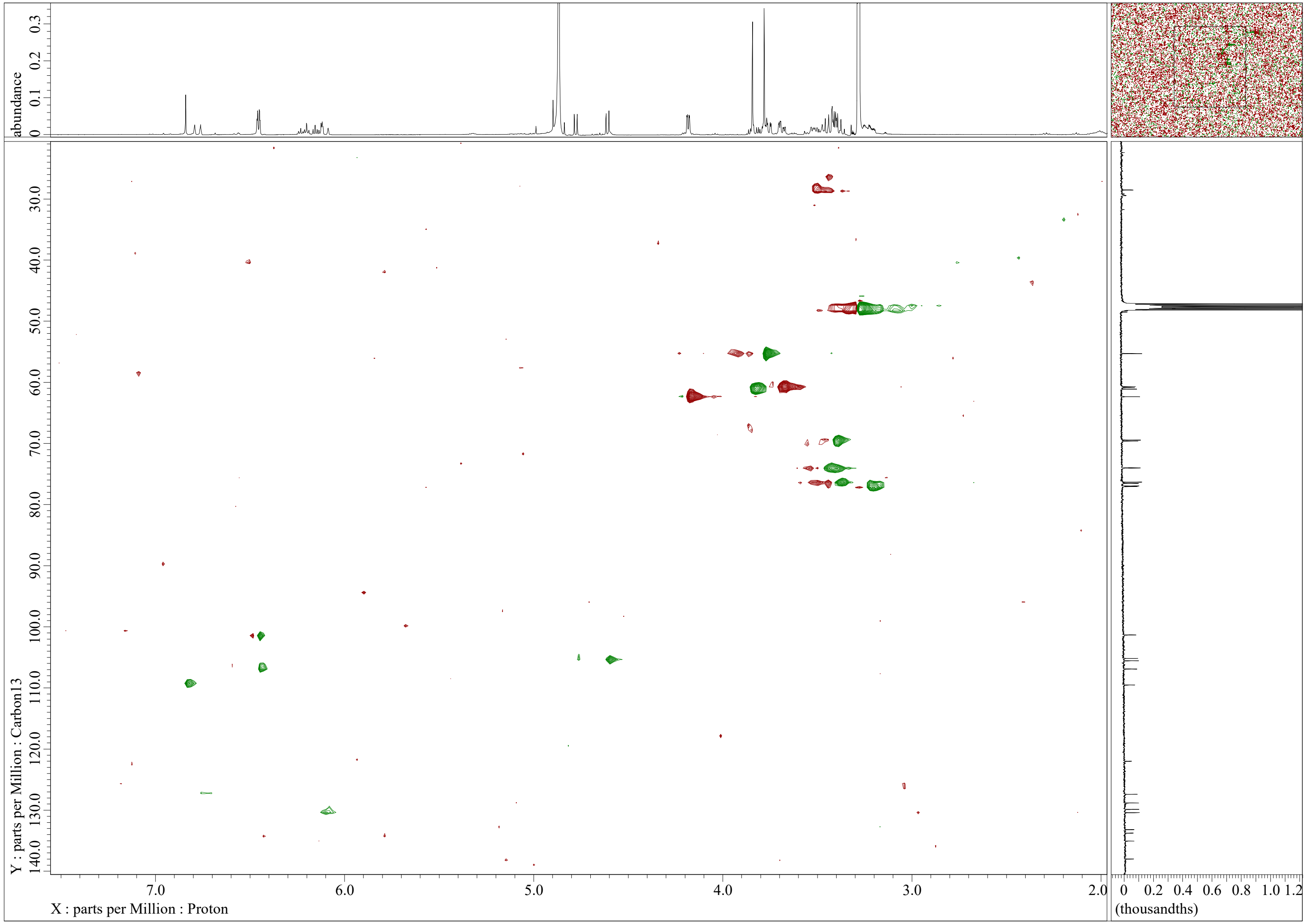

Figure S11. The COSY spectrum of compound **2** in CD_3OD

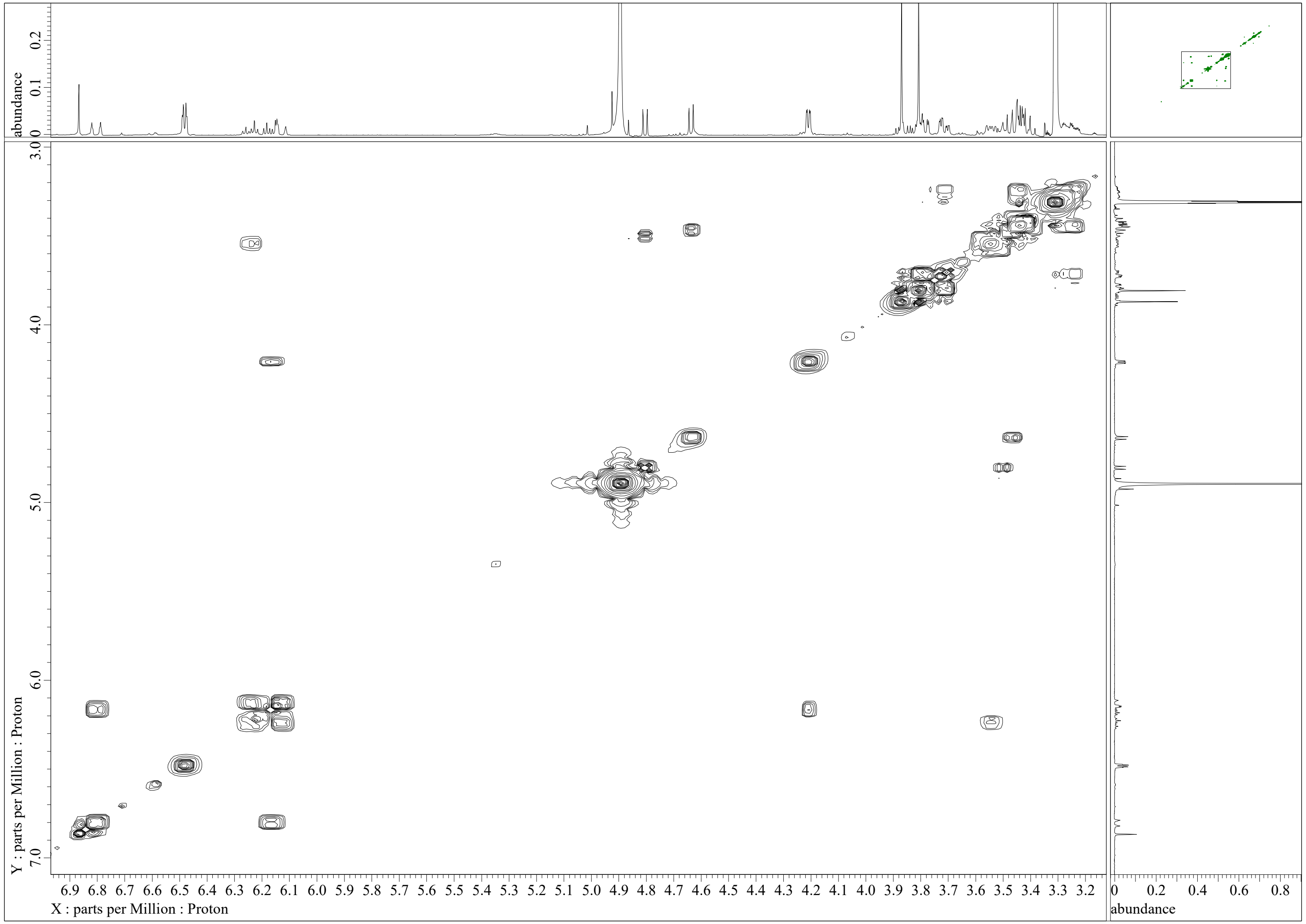

Figure S12. The HMBC spectrum of compound **2** in CD_3OD

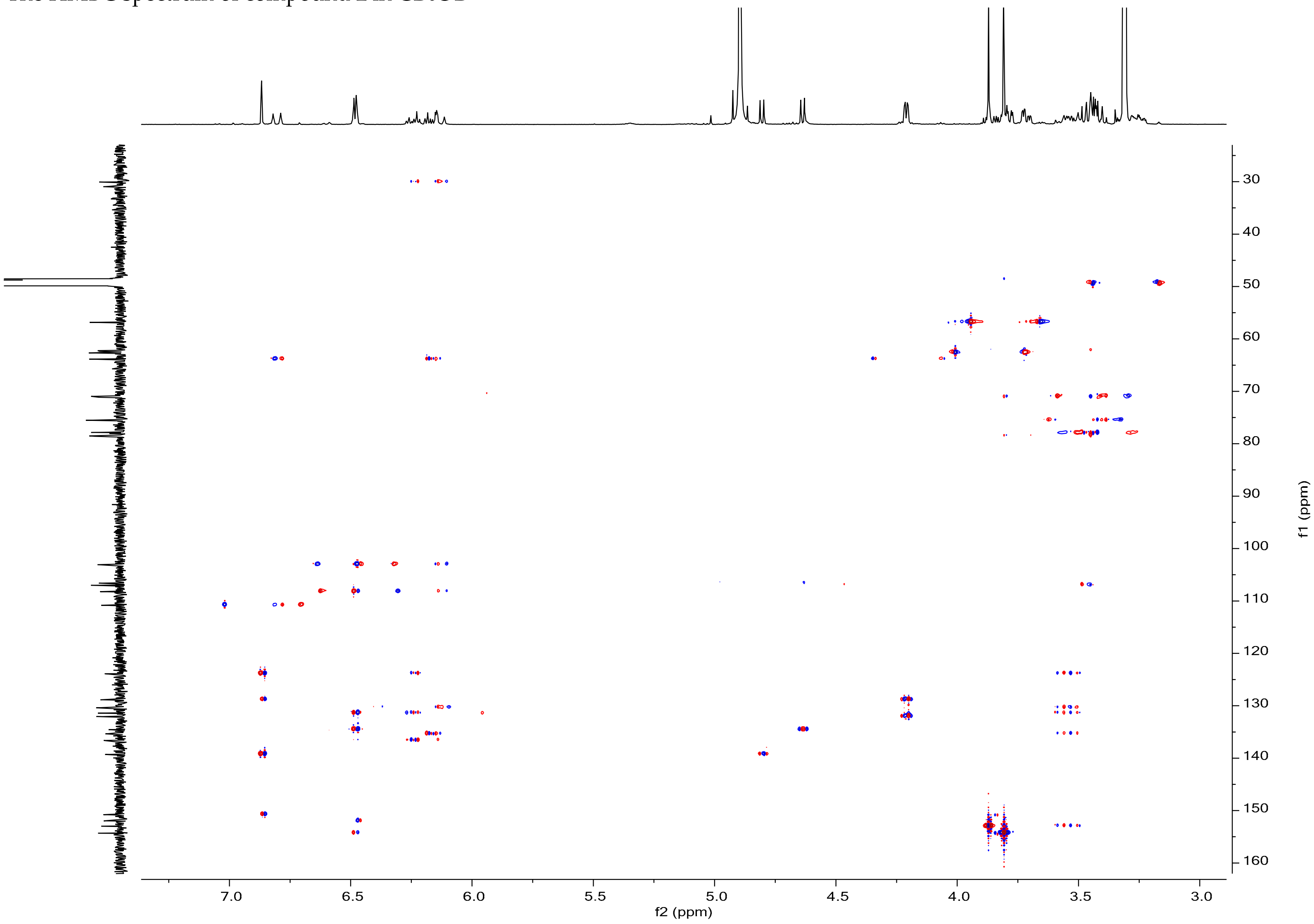

Figure S13. HR-Q-TOF -MS spectrum of compound **3**

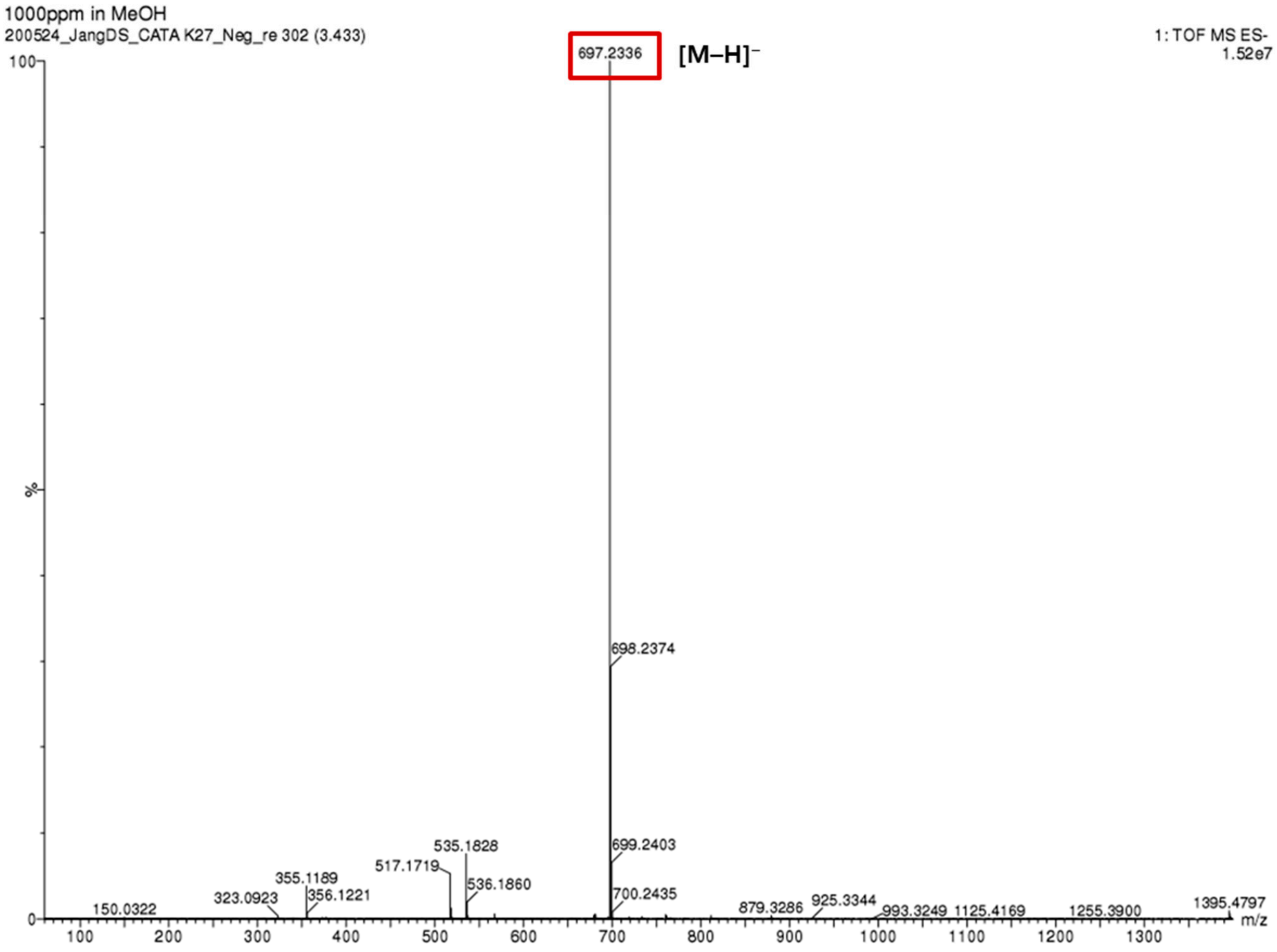

Figure S14. The ^{1}H-NMR (500 MHz, CD_3OD) spectrum of compound **3**

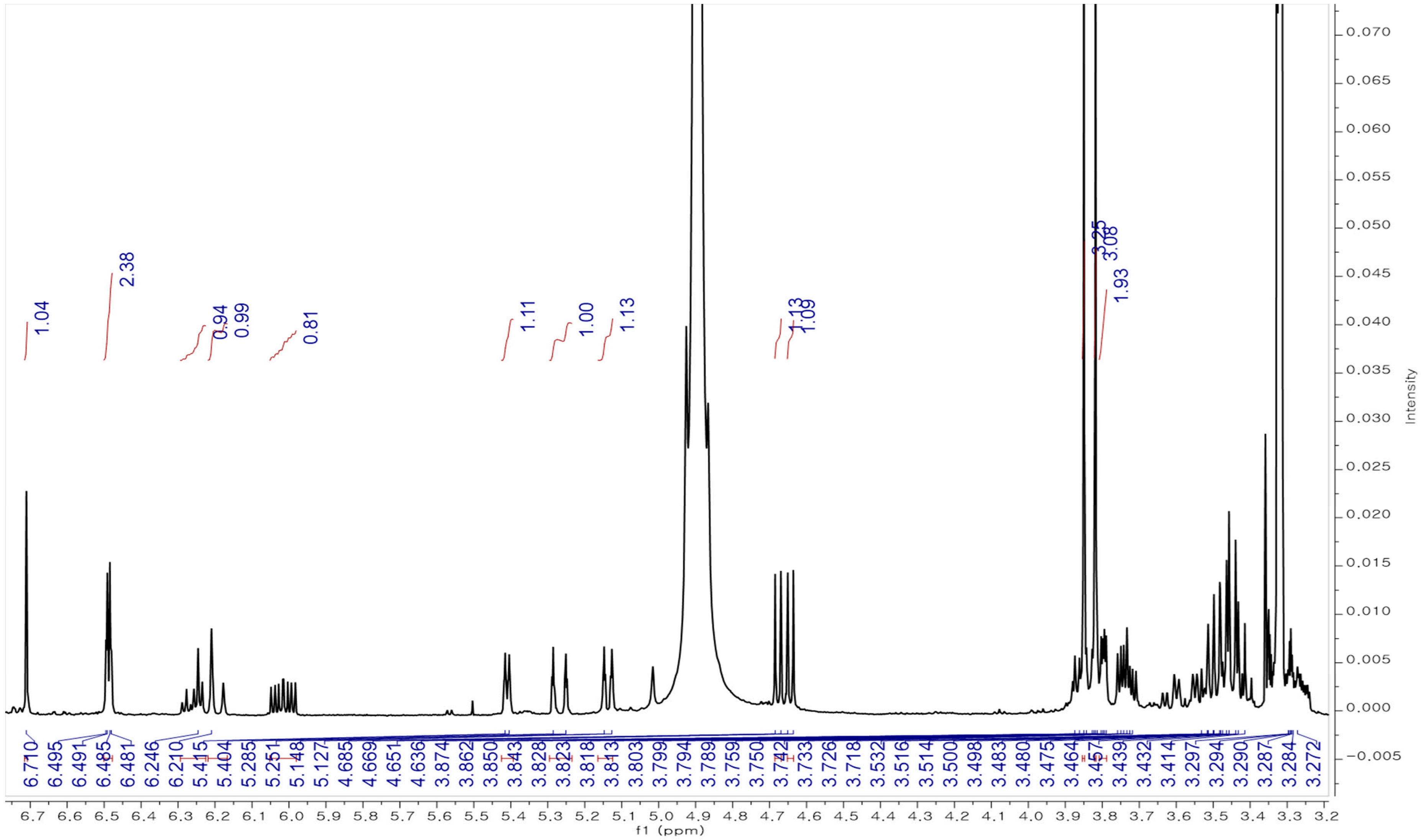

Figure S15. The ^{13}C-NMR (125 MHz, CD_3OD) spectrum of compound **3**

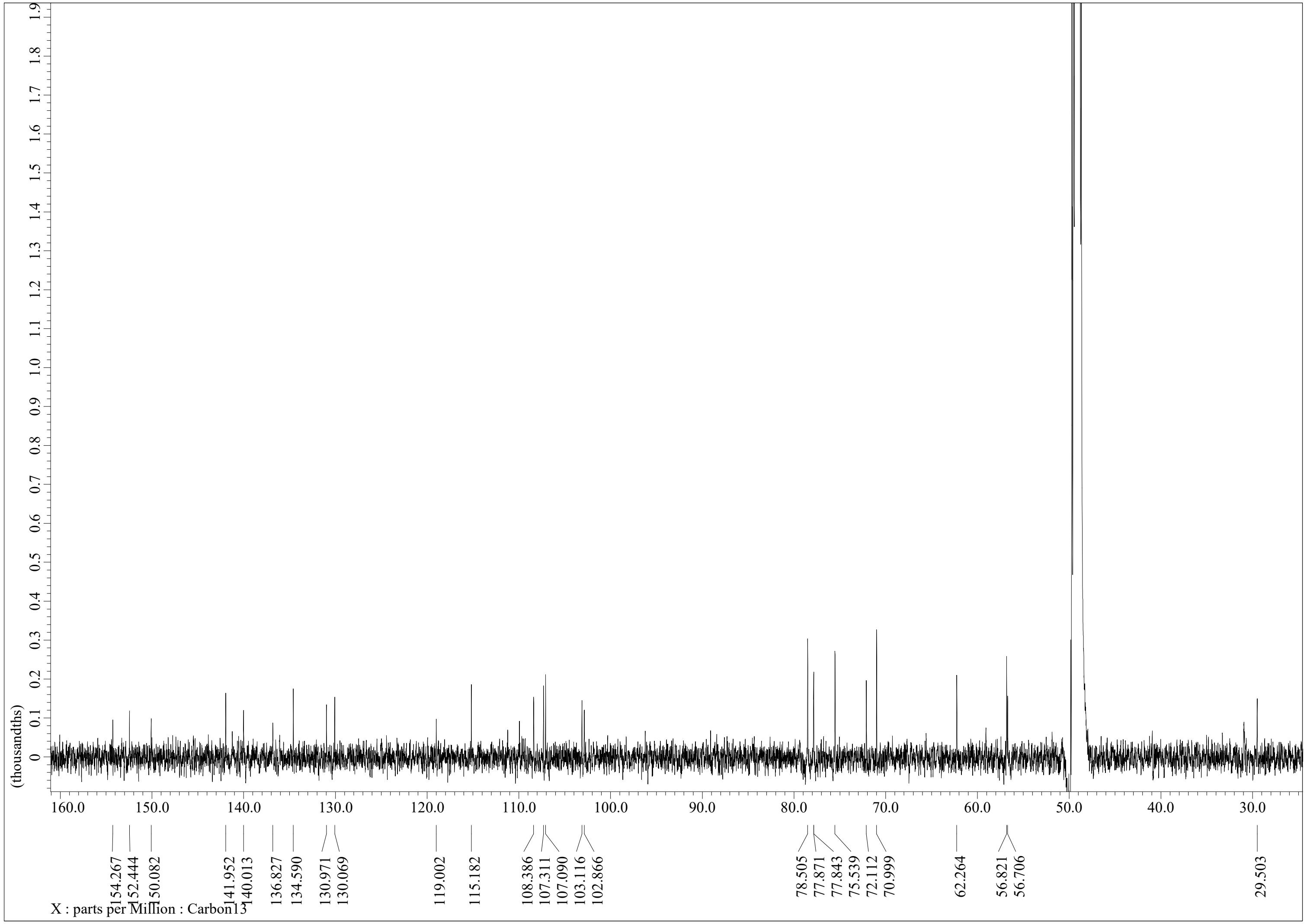

Figure S16. The HSQC spectrum of compound **3** in CD_3OD

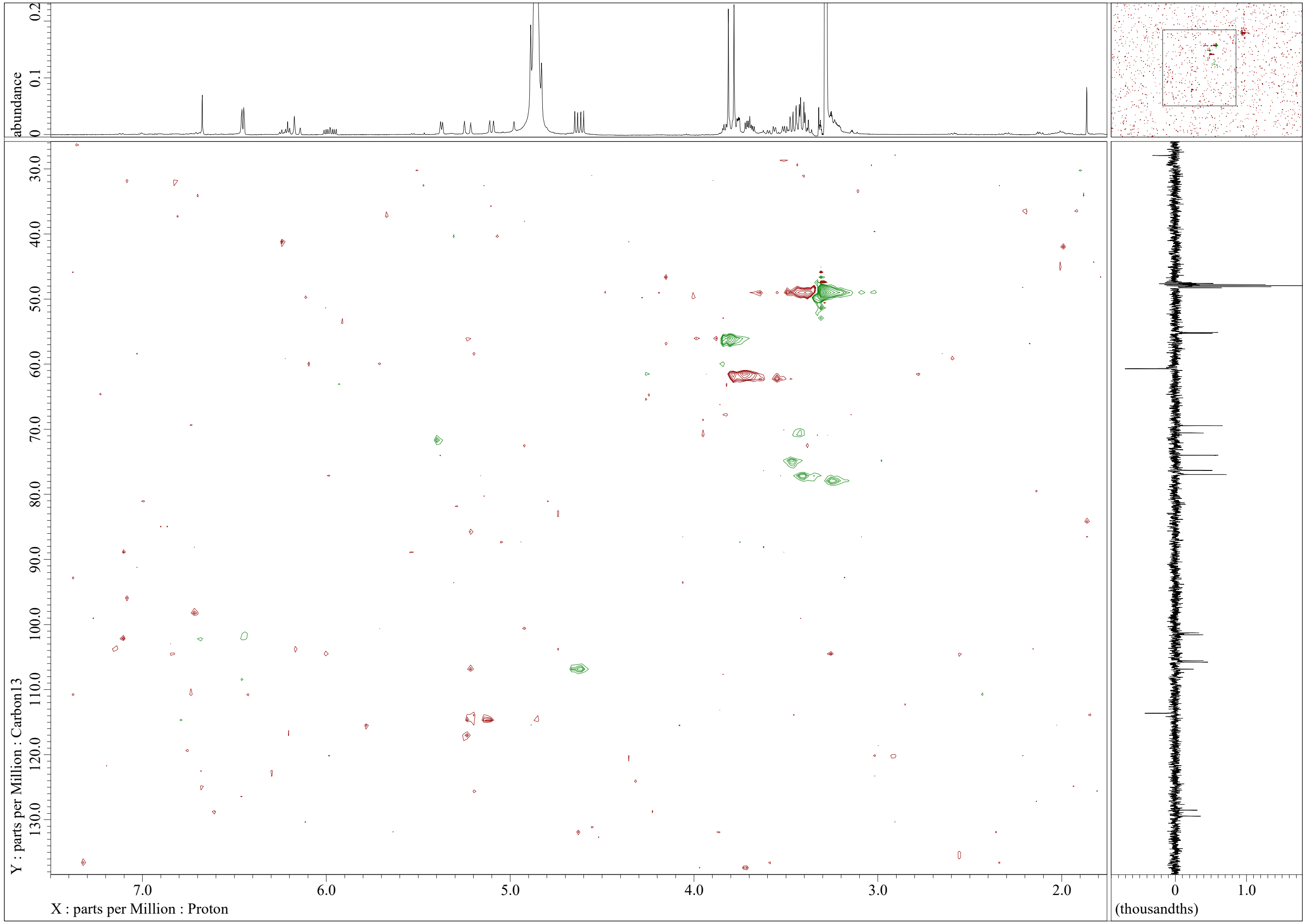

Figure S17. The COSY spectrum of compound **3** in CD_3OD

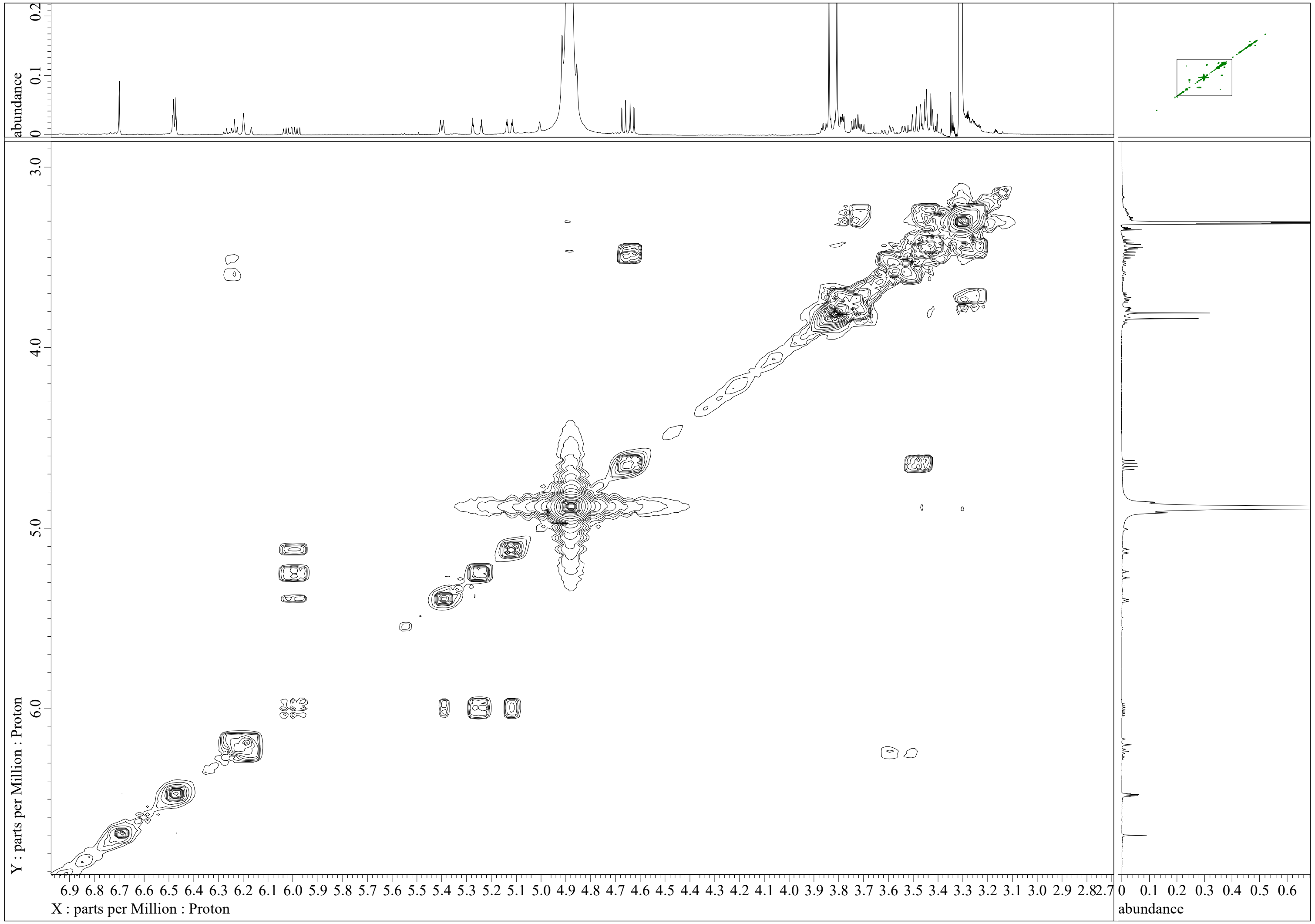

Figure S18. The HMBC spectrum of compound **3** in CD_3OD

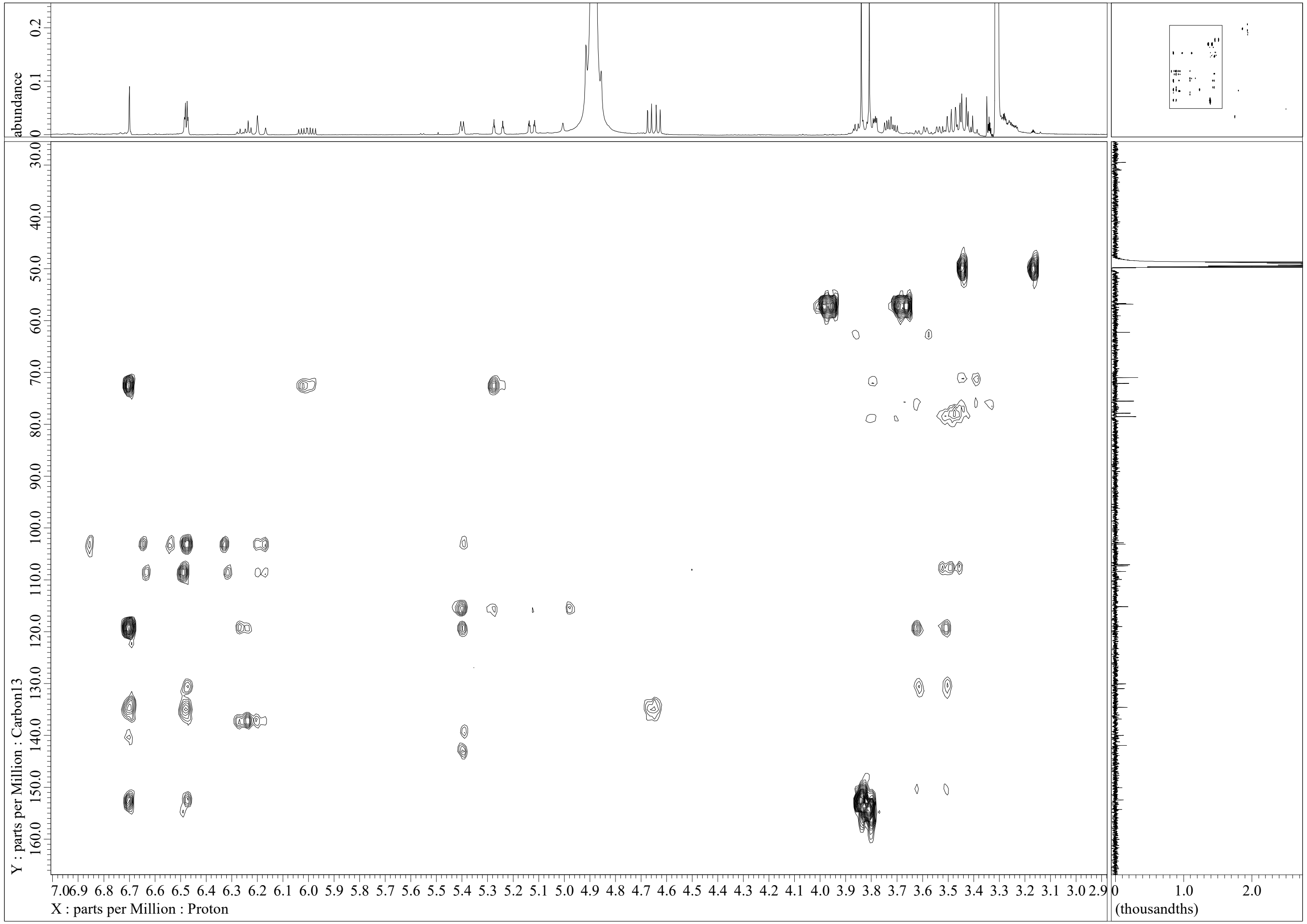

Supplement: Supplementary file 1 [file plants-09-01232-s001.pdf]
